# Physical Inactivity and Metabolic Syndrome in the United Arab Emirates: A Systematic Review and Meta-analysis of Parallel Epidemics

**DOI:** 10.1007/s40279-026-02493-6

**Published:** 2026-07-22

**Authors:** Bushra Ali, Chris Bishop, Tim Evans, Anne Elliott

**Affiliations:** 1 London Sport Institute, Faculty of Science and Technology, Middlesex University, London, UK; 2 Business and Political Economy, Middlesex University London, London, UK

## Abstract

**Background:**

Metabolic syndrome (MetS), a cluster of cardiovascular risk factors strongly associated with physical inactivity, has emerged as a critical global public health concern. The United Arab Emirates (UAE) exhibits elevated prevalence rates of both conditions, driven by rapid urbanization and lifestyle transitions. However, a comprehensive synthesis of the epidemiological evidence remains lacking.

**Objective:**

This systematic review and meta-analysis evaluated the prevalence of physical inactivity and MetS in the UAE general population.

**Methods:**

Data were obtained from systematic searches of eight databases (CINAHL, Embase, Medline, PsycINFO, Scopus, Web of Science, Cochrane Library, SPORTDiscus). Inclusion criteria encompassed observational studies reporting prevalence data on physical inactivity (primarily defined as not meeting World Health Organization (WHO) guidelines: ≥ 150 min/week moderate-intensity activity) and/or MetS in UAE adults. Sixty prevalence studies were included, with data pooled using random-effects meta-analysis.

**Results:**

Three meta-analyses estimated physical activity (PA) prevalence using different methods across 39 studies (*n* = 77,360), yielding a pooled prevalence of 32.2% (95% CI 24.8–40), substantially lower than the WHO global average of 69%. Accelerometer data (two studies, *n* = 296) showed 23.5% (95% CI 18.8–28.5), while survey-based reports (11 studies, *n* = 18,034) indicated 51.1% (95% CI 37.9–64.3). MetS prevalence was 31.2% (95% CI 23.6–39.4) from 14 studies (*n* = 22,835). Both conditions showed high heterogeneity and sex-related differences, with lower PA and distinct MetS patterns in women. Physical inactivity was associated with 34% increased odds of MetS.

**Conclusion:**

Physical inactivity and MetS represent important public health priorities in the UAE, with notable demographic variations warranting targeted interventions. Building on existing government health initiatives, addressing this burden requires comprehensive, multisectoral interventions encompassing urban environmental redesign, culturally adapted health promotion programmes, and context-specific diagnostic frameworks tailored to the UAE’s distinct environmental, sociocultural, and economic context.

**Supplementary Information:**

The online version contains supplementary material available at https://doi.org/10.1007/s40279-026-02493-6.

## Key Points

**Table:** 

Physical inactivity remains highly prevalent in the United Arab Emirates (UAE), with consistently low activity levels documented across three decades and measurement methods.
Metabolic syndrome (MetS) affects roughly one-third of adults, reflecting a substantial and persistent metabolic health burden nationally.
Demographic inequalities, particularly by age, sex, and nationality, shape both inactivity and metabolic risk, highlighting priority groups for intervention.
The relationship between physical inactivity and MetS is clear, with inactivity associated with 34% higher odds of MetS, reinforcing the need for integrated prevention strategies.
Harmonizing measurement and diagnostic approaches is essential to strengthen surveillance, improve comparability, and guide targeted public health action.

## Introduction

The United Arab Emirates (UAE) government has undertaken substantial efforts to improve population health through various strategic initiatives, including the implementation of comprehensive national health surveys [1–3], development of the National Agenda for Health and Wellbeing [4], and significant investment in healthcare infrastructure and preventive medicine programs [5]. These efforts have successfully increased health awareness, established robust disease surveillance systems, and enhanced healthcare accessibility across the Emirates [5]. However, like many rapidly developing nations undergoing significant lifestyle transitions, the UAE continues to face challenges with physical inactivity [4, 6] and the metabolic syndrome (MetS) [7, 8], conditions that underpin much chronic disease in the region and require sustained, focused attention alongside existing health promotion efforts.

Noncommunicable diseases (NCDs), including cardiovascular diseases, type 2 diabetes mellitus, and stroke, represent a growing public health concern globally [9] and are particularly pressing in high-income, rapidly urbanizing countries like the UAE [8, 10]. A key contributor to this burden is MetS, a cluster of interrelated metabolic abnormalities (central obesity, hypertension, hyperglycaemia, and dyslipidaemia) [11] that together elevate the risk of chronic disease and premature mortality. Physical inactivity is one of the most important behavioural risk factors underlying the development of MetS [12, 13] and, more broadly, NCDs. Sedentary lifestyles, increasingly prevalent in urban settings, play a significant role in shaping the metabolic health profile of populations and are a growing concern in the UAE context.

In recent years, several population-based studies have examined the prevalence of physical inactivity and MetS across different regions and demographic groups in the UAE [8, 14]. However, reported prevalence rates vary substantially between studies (physical inactivity: approximately 33–85%; MetS: approximately 7–50%), reflecting differences in sampling strategies, studied populations, and research designs. This variation has made it difficult to estimate the national burden of these conditions with confidence. While some reports suggest that physical inactivity may affect a large proportion of the adult population [10], others highlight rising MetS rates among both Emiratis and expatriate residents [8]. Despite the growing number of studies, there remains uncertainty regarding the scale of the problem and its distribution across different groups.

To date, there has been no systematic synthesis of the evidence on the prevalence of physical inactivity and MetS specifically within the UAE only. Existing reviews have primarily examined PA [15], with no systematic review to date investigating MetS among adults in the UAE. Examining physical inactivity and MetS jointly is more appropriate because physical inactivity is a key modifiable behavioural risk factor that directly contributes to the development and progression of MetS through metabolic, cardiovascular, and inflammatory pathways [16]. Studying both conditions together allows for a more integrated understanding of their co-occurrence and interaction, which is crucial for designing holistic public health interventions tailored to the UAE’s unique demographic and lifestyle characteristics. In addition, little is known about how prevalence varies by key demographic variables such as age, sex, and nationality, or by geographic location within the UAE. Temporal trends also remain largely unexamined, limiting our understanding of how these epidemics may be evolving. Without clarity on these aspects, efforts to design effective public health interventions and allocate resources appropriately to combat such issues may be compromised.

With this in mind, this systematic review and meta-analysis aimed to: (1) determine the current prevalence of physical inactivity and MetS in UAE populations; (2) investigate how these prevalence rates vary across demographic subgroups (age, sex, nationality) and geographic regions within the UAE; (3) examine the temporal changes in prevalence over time; and (4) critically evaluate the measurement approaches and diagnostic criteria used in existing UAE-based studies. Operational definitions of key terms used throughout this review are provided in Table 1.

**Table 1:** Operational definitions for key terms in this systematic review

Terminology	Operational definition
Physical inactivity	Failure to meet established PA guidelines or recommendations. The most commonly applied threshold across included studies was the WHO recommendation of ≥ 150 min per week of moderate-intensity activity or ≥ 75 min per week of vigorous-intensity activity. Studies varied in specific definitions, including: self-reported engagement in “no PA whatsoever” during a specified recall period, activity levels below study-specific thresholds (e.g., < 30 min daily walking), categorical classifications based on validated questionnaires (IPAQ, GPAQ), or objective accelerometer data indicating insufficient moderate-to-vigorous PA. The term “physically inactive” is used throughout this review to describe individuals not meeting these guideline thresholds
Sedentary behaviour	Waking activities characterized by low energy expenditure (≤ 1.5 metabolic equivalents (METs)) while in a sitting, reclining, or lying posture. This is conceptually distinct from physical inactivity; an individual can meet activity guidelines yet still engage in high levels of sedentary behaviour throughout the day. Studies using objective measurement (accelerometry) typically quantified sedentary time as total sitting/lying hours per day during waking hours
Insufficient physical activity	Activity levels below recommended guidelines but above complete inactivity. This encompasses individuals engaging in some PA that does not meet the duration, frequency, or intensity thresholds established for optimal health benefits (e.g., 100 min/week instead of the recommended 150 min/week)
Metabolic syndrome	A cluster of interrelated metabolic abnormalities that together elevate the risk of cardiovascular disease and type 2 diabetes. Diagnostic criteria varied across included studies *ATP III* (Adult Treatment Panel III) requires three of five components: elevated waist circumference (≥ 102 cm men, ≥ 88 cm women), elevated triglycerides (≥ 150 mg/dL), reduced high-density lipoprotein cholesterol (< 40 mg/dL men, < 50 mg/dL women), elevated blood pressure (≥ 130/85 mmHg), and elevated fasting glucose (≥ 100 mg/dL) *IDF* (International Diabetes Federation) mandates central obesity (ethnicity-specific waist thresholds: ≥ 94 cm Middle Eastern men, ≥ 80 cm Middle Eastern women) plus two of four additional factors *Harmonized criteria* require any three of five components with ethnicity-specific adaptations. These variations can substantially affect prevalence estimates by 5–15 percentage points
Physical activity measurement methods	Included studies employed diverse approaches: *Objective measures* included accelerometry (activPAL, ActiGraph) providing continuous data on activity intensity with standardized cut-points, used in four studies (*n* = 437) *Validated questionnaires* such as IPAQ and GPAQ assessed self-reported activity across multiple domains with established scoring protocols, used in nine studies (*n* = 14,349) *Simple self-report measures* ranged from single dichotomous questions to brief surveys with varying recall periods and validation status, used in 41 studies (*n* = 83,073). This methodological heterogeneity necessitated separate meta-analyses by measurement approach

*PA* physical activity, *WHO* World Health Organization, *IPAQ* International Physical Activity Questionnaire, *GPAQ* Global Physical Activity Questionnaire, *MET* Metabolic Equivalent of Task

## Methods

### Study Design

The literature pertaining to the prevalence of physical inactivity and MetS within the adult population of the UAE was systematically identified and synthesized in accordance with PRISMA guidelines [17]. The review protocol was registered with PROSPERO (CRD420251065730) and later amended (Amendment v2.0, 7 October 2025) to refine the review focus from association analysis to prevalence synthesis in alignment with Joanna Briggs Institute (JBI) methodology (see Online Supplementary Material (OSM) Document 1 for details).

### Eligibility Criteria

Observational study designs were eligible for inclusion, including cross-sectional, cohort, and case–control studies. This inclusive approach was chosen to capture the full breadth of available evidence while acknowledging that cross-sectional designs limit causal inference. Qualitative studies (e.g., interviews, focus groups, ethnographic studies) were also considered eligible. Studies that exclusively evaluated intervention effects without reporting baseline prevalence data were excluded.

#### Population

Studies were included if they reported data on adults aged 18 years and older who were residents of the UAE, including both Emirati nationals and expatriate populations. No restrictions were placed on sex, nationality, ethnicity, socioeconomic status, or geographic location within the seven Emirates. Studies conducted in both urban and rural settings were eligible, as were those examining specific population subgroups (e.g., general population, working adults, university students), provided participants were UAE residents. Studies focusing exclusively on populations outside the UAE or on children and adolescents under 18 years were excluded.

#### Outcomes

Studies were required to report prevalence data on physical inactivity, MetS, or both conditions. For physical inactivity, eligible studies included those using quantitative measures such as validated questionnaires (e.g., International Physical Activity Questionnaire (IPAQ), Global Physical Activity Questionnaire (GPAQ)), objective measures (e.g., accelerometers, pedometers, heart rate monitors), or quantified PA levels (e.g., minutes per week, metabolic equivalent of task (MET)-minutes, step counts). Binary or categorical measures of PA status were also eligible, including self-reported dichotomous classifications (“active” vs. “inactive” or “sedentary”), single-item questions, and categorical classifications. Studies using any definition or cut-off for classifying individuals as physically active or inactive were included, regardless of validation status, with quality and comparability assessed during risk-of-bias evaluation and addressed in sensitivity analyses. For MetS, studies were required to report prevalence data using established diagnostic criteria including the Adult Treatment Panel III (ATP III), International Diabetes Federation (IDF), World Health Organization (WHO), or harmonized criteria (a 2009 consensus definition reconciling the IDF and AHA/NHLBI criteria, requiring three of five components with ethnicity-specific thresholds). Studies focusing solely on other health conditions without assessing physical inactivity or MetS prevalence were excluded, as were intervention-only studies that did not report baseline or habitual physical inactivity levels or examine associations between inactivity and MetS.

### Information Sources and Search Strategy

A comprehensive, three-step search strategy was employed to identify relevant published and unpublished studies on physical inactivity and MetS in the UAE on 21 June 2025, minimizing publication bias. Peer-reviewed studies were identified through systematic searches of eight electronic databases: CINAHL, Embase, Medline, PsycINFO, Scopus, Web of Science, Cochrane Library, and SPORTDiscus. Grey literature was identified through: (1) thesis databases (ProQuest Dissertations and Theses, UAE university institutional repositories including UAE University, Zayed University, and American University of Sharjah); (2) conference proceedings (Conference Proceedings Citation Index via Web of Science); and (3) government health surveillance reports (UAE Ministry of Health and Prevention, Dubai Health Authority, Abu Dhabi Department of Health). Hand-searching of reference lists from included studies and relevant systematic reviews supplemented these searches. Search terms were derived from medical subject headings and synonyms related to physical inactivity, MetS, and the UAE. No date restrictions were applied at search stage. Only English-language publications were eligible for inclusion. Search strings were developed using identified terms combined with Boolean operators. An example search string for one of the databases is provided in Table 2. The complete search strategies for all databases and grey literature sources are provided in OSM Document 2.

**Table 2:** Medline search strategy

Concept	Search terms
Physical inactivity	“physical activity” OR “physical inactivity” OR exercise OR sedentary OR workout
Metabolic syndrome	“metabolic syndrome” OR “syndrome X” OR “deadly quartet” OR “cardiometabolic”
Prevalence/epidemiology	“prevalence” OR “incidence” OR “epidemiology” OR “rate” OR “occurrence” OR “risk factor*”
Location (UAE)	“united arab emirates” OR UAE OR “Abu Dhabi” OR Dubai OR Sharjah OR Ajman OR “Umm Al Quwain” OR “Ras Al Khaimah” OR Fujairah OR “Al Ain”

Search executed in Medline via the EBSCO platform on 21 June 2025. Two parallel searches were conducted: (1) Physical Inactivity AND Prevalence/Epidemiology AND Location, yielding 586 records; and (2) Metabolic Syndrome AND Prevalence/Epidemiology AND Location, yielding 274 records. Quotation marks indicate phrase searches; the asterisk (*) denotes truncation. Limits applied: English language

Similar strategies, adapted for respective database syntax and controlled vocabularies, were used for other platforms. Search filters included English language studies published up to June 2025.

### Methodological Quality Assessment

The methodological quality of these studies was appraised using the JBI Critical Appraisal Checklist for Studies Reporting Prevalence Data within JBI SUMARI.

### Data Extraction

A standardised data extraction form was developed and pilot-tested to ensure consistency and accuracy across studies. Relevant information was independently extracted by two reviewers, including study characteristics (author, year, study design, setting), sample demographics (age, sex, nationality), diagnostic criteria for MetS (ATP III, IDF, WHO, or harmonized definitions), measurement tools for PA (IPAQ, GPAQ, accelerometry, pedometers, or self-report), and reported prevalence estimates for both conditions. Additional variables captured included geographic region, socioeconomic indicators, and risk factor distribution. Discrepancies were resolved through discussion or consultation with a third reviewer. Where individual studies reported multiple prevalence estimates (e.g., stratified by sex or age, multiple diagnostic criteria, or multiple time points), a standardized selection hierarchy prioritizing overall population estimates, WHO-standard activity thresholds, and crude prevalence was applied to ensure consistency.

### Data Analysis

The extracted data from eligible studies were pooled using statistical proportional meta-analysis in JBI SUMARI. Prevalence estimates for MetS and physical inactivity were calculated by applying the Freeman-Tukey double arcsine transformation to stabilise variances of proportions across included studies. All analyses were conducted using the random-effects model to account for underlying population and study differences. For each outcome, heterogeneity between studies was assessed with the *I*^2^ statistic and its corresponding *p*-value. Mixed-effects models with 95% confidence intervals were used for pooled proportions. For studies with multiple groups, results from the largest group were selected for synthesis. Publication bias assessment was not performed via Egger’s test or funnel plots, as their utility for prevalence meta-analysis is limited and could be misleading due to selection bias. Leave-one-out sensitivity analyses were performed for each meta-analysis where the number of contributing studies permitted, by sequentially excluding each study and recalculating the pooled estimate to assess the influence of individual studies on the overall result [18]. Four separate forest plots were generated: one for MetS prevalence, one for PA prevalence (IPAQ/GPAQ-based studies), one for accelerometer-based data and another for self-reported PA using other methods. Each forest plot displays study-level proportions and the overall pooled estimate with corresponding 95% confidence intervals.

## Results

### Study Selection

**Fig. 1:**
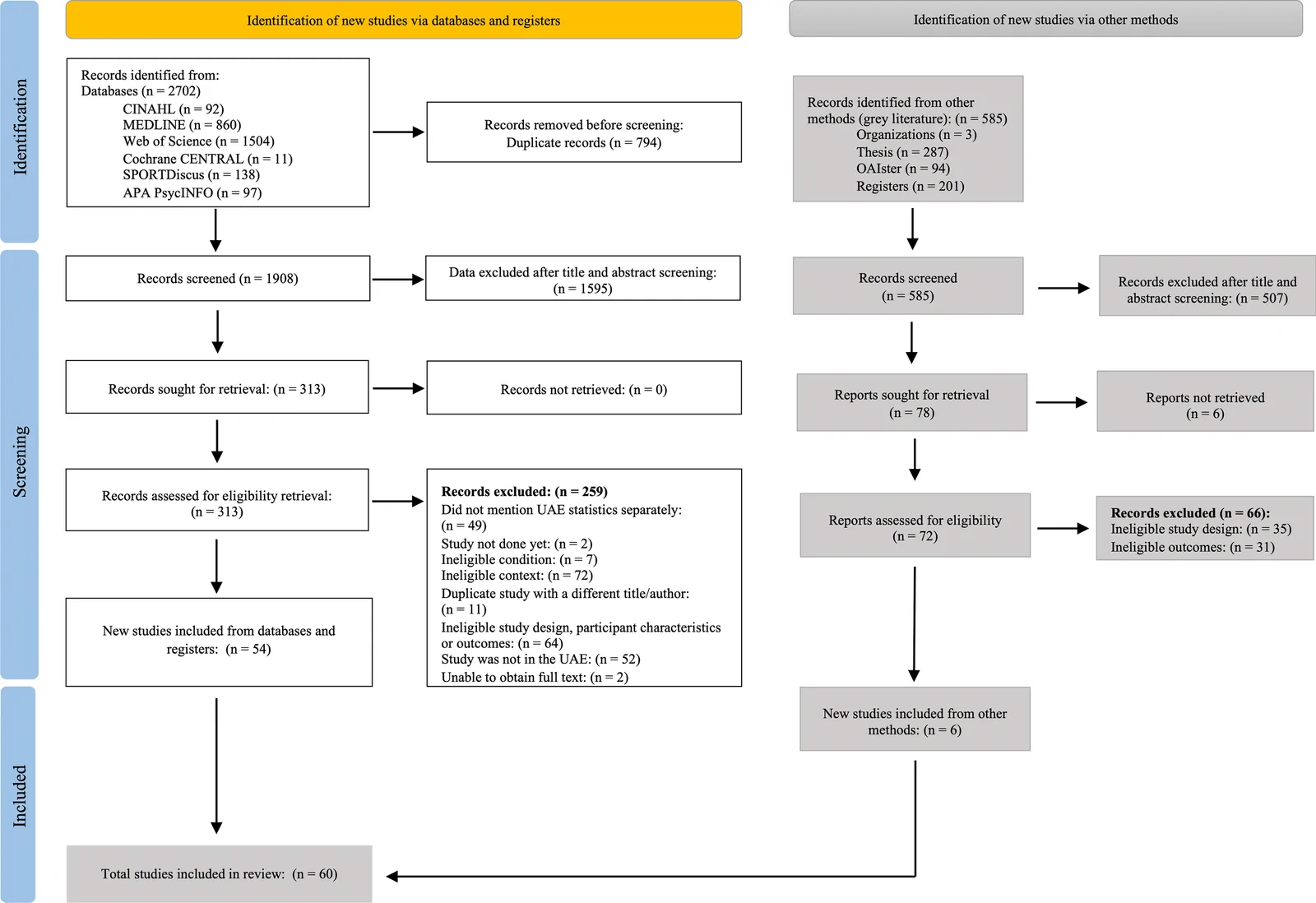
PRISMA flow diagram

The search of academic databases and grey literature sources identified a total of 3287 records: 2702 from electronic databases (CINAHL, *n* = 92; MEDLINE, *n* = 860; Web of Science, *n* = 1,504; Cochrane CENTRAL, *n* = 11; SPORTDiscus, *n* = 138; and APA PsycINFO, *n* = 97) and 585 from other sources, comprising organizations (*n* = 3), theses (*n* = 287), OAIster (*n* = 94), and registers (*n* = 201). Following the removal of 794 duplicate records from the database results, 1,908 records were screened by title and abstract, of which 1,595 were excluded. The remaining 313 records were all sought for retrieval and obtained in full text for eligibility assessment. Of these, 259 were excluded for the following reasons: no UAE-specific statistics reported (*n* = 49); registered study protocols for incomplete or ongoing studies without published results (*n* = 2); ineligible condition (*n* = 7); ineligible context (*n* = 72); duplicate study with a different title or author (*n* = 11); ineligible study design, participant characteristics, or outcomes (*n* = 64); study not conducted in the UAE (*n* = 52); and full text unobtainable (*n* = 2). This yielded 54 eligible studies from the database search. Of the 585 records identified through other methods, 507 were excluded after title and abstract screening, and 78 reports were sought for retrieval; six could not be obtained. Of the 72 retrieved reports, 66 were excluded, 35 for an ineligible study design and 31 for ineligible outcomes, leaving six reports that were assessed as eligible and included. In total, 60 studies (54 from databases and registers and six from other methods) were included in the systematic review (Fig. 1).

### Study Characteristics

The 60 included studies comprised 14 examining MetS prevalence, 39 investigating self-reported PA, 11 employing validated survey instruments for PA assessment, and two using accelerometer-based data. Six studies reported data on both MetS and PA, and hence category totals exceed the overall number of included studies. A comprehensive overview of extracted data from included studies is presented in Tables 3, 4, 5, 6, which respectively address: MetS prevalence (Table 3), self-reported PA levels in the UAE (Table 4), characteristics of studies using validated surveys to assess self-reported PA or inactivity (Table 5), and characteristics of studies employing accelerometry to measure PA in the UAE (Table 6).

**Table 3:** Characteristics of included studies on the prevalence of the metabolic syndrome

Study	Sample (no., sex, age)	Design	MetS (criteria and prevalence)	Physical activity measures and classification criteria	Physical activity prevalence	Notes
Al-Sarraj et al. [19]	*n* = 309, 98 (M), 215 (F), 38.3 ± 14.6 y	CS	ATPIII (25.2%)	Any exercise in previous 2 weeks	34%	
Malik and Razig [20]	*n* = 4,097, 1,691 (M), 2,406 (F), 20–60 + y	CS	ATPIII (39.6%); IDF (40.5%)	At least 30 min of brisk walking daily during previous month	33.5%	
Baynouna et al. [21]	*n* = 817, 18–60 + y	CS	ATPIII (37.6%)			
Al-Sarraj et al. [22]	*n* = 227, 74 (M), 153 (F), 18–50 y	CS	IDF (40.5%)	GPAQ	6%	
Hajat and Shather [7]	*n* = 760, 325 (M), 435 (F), 18 + y	CS	IDF (48.7%), ATPIII (50.3%)			
Shehab et al. [23]	*n* = 1,259, 885 (M), 374 (F), 22–100 y	CS	IDF & AHA/NHLBI 67.6%			
Alkaabi et al. [24]	*n* = 110, F, 30–65 y	CS	IDF (45%)	≥ 150 min/wk	15%	
Al Dhaheri et al. [25]	*n* = 555, F, 17–25 y	CS	IDF/AHA/NHLBI harmonized criteria (6.8%)			
Awadallah et al. [26]	*n* = 172, 85 (M), 87 (F), 20–60 y	CS	IDF (22.1%)			49.4% had large waist circumference
Mezhal et al. [27]	*n* = 5,161, 3,196 (M), 1,965 (F), 18–40 y	CS	ATPIII (18%)			
Mahmoud and Sulaiman [8]	*n* = 3,212, 2,380 (M), 832 (F), 18–60 y	CS	ATPIII (37.4%)	Self-reported PA (Yes/No)	14.9%	
AlMuhairi [28]	*n* = 5,050, 3,044 (M), 2,006 (F), 18–40 y	CS	ATPIII (14.6%), IDF (19.3%), JIS (22.4%)	GPAQ	68.4% active from the sample (3438) who answered	Leisure inactivity has 1.70–1.76 × higher odds of overall MetS
Harfil et al. [29]	*n* = 272, 107 (M), 165 (F), 18–55 y	CS	IDF (25%)			
Aziz et al. [30]	*n* = 833	CS	AACE (28.6%)			

*CS* cross-sectional survey, *GPAQ* General Physical Activity Questionnaire, *M* male, *F* female, *ATPIII* Adult Treatment Panel III, *IDF* International Diabetes Federation, *AACE* American Association of Clinical Endocrinologists, *AHA/NHLBI* The American Heart Association (AHA) and the National Heart, Lung, and Blood Institute

**Table 4:** Extracted data from studies assessing self-reported physical activity levels in the UAE

Study	Sample (no., sex, age)	Design	Physical activity measures and classification criteria	Physical activity prevalence	Notes
Amine and Samy [31]	*n* = 566 (F), 18–22 + y	CS	Self-reported questionnaire asking about physical exercise practice and severity; categorized as “mild exercise” (walking on campus) vs. “no exercise”	4.4%	PA assessment very limited (only walking vs. none)
Sabri et al. [32]	*n* = 872, 460 (M), 412 (F), 20–65 y	matched case–control	Self-reported questionnaire; classified as “No activity” (< 1 h/week) “Active” (≥ 1 h/week of sport)	47.8%	
Carter et al. [33]	*n* = 535 (F), 20–79 y	CS	Validated questionnaire for elderly	84%	Prevalence is inflated by the elderly-focused survey method
DHHS [1]	*n* = 20,000, 10,000 (M), 10,000 (F), 18–60 + y	CS	Self-report: weekly minutes of moderate/vigorous PA ≥ 10 min bouts (based on CDC/ACSM 2008 guidelines: ≥ 150 min moderate or ≥ 75 min vigorous PA per week)	19%	
Saadi et al. [34]	*n* = 555, 79 (F), 315 (F), 38–54 y	CS	Self-reported walking for exercise	14%	
Muttappallymyalil et al. [35]	*n* = 110, 26 (M), 84 (F)	CS	30-day recall self-administered questionnaire assessing time spent in various moderate and vigorous PA	90% engaged in some form of regular PA; 10% completely inactive	
Hussain and Hassan [36]	*n* = 2,226, 18–65 y	CS	Self-reported practice of regular PA	34.6%	PA was significantly higher among Emirates nationals, highly educated individuals (university +), and high-income earners (≥ 10,000 ED)
Khan et al. [37]	*n* = 168, 47 (M), 121 (F), 18–24 y	CS	Self-reported exercise frequency (≥ 150 min/week considered regular)	27%	
Alsafar et al. [38]	*n* = 741, 470 (M), 271 (F), 18 + y	CS	Self-reported questionnaire assessing whether participants performed daily PA (minimum 30 min walking/day)	54.7%	
Trainer [39]	*n* = 103 (F),18–30 y	CS	24-h activity recall (minimum 20 min exercise definition—walking, sport, gym, etc.)	~ 30%	
Hashim et al. [40]	*n* = 733, 445 (M), 288 (F),22–70 y	CS	Self-reported questionnaire asking about regular exercise	39%	
Stefanopoulou et al. [41]	*n* = 372 (F), 45–55 y	CS	Exercise frequency (any exercise and strenuous exercise)	11% exercised 2–7 times/week; 43.1% ≤ 1/week; 45.9% rarely/never	
Katsaiti and El Anshasy [42]	*n* = 915, 327 (M), 588 (F),17–35 y	CS	Self-reported exercise hours per week (scale 0–4); also assessed sedentary behaviours (TV hours, PC hours)	11.5%	
Hoffman [43]	*n* = 340 (M), 18–29 y	CS	Self-reported PA levels: rare, light, moderate, heavy; gym attendance frequency	56.7%	
Sulaiman et al. [44]	*n* = 3,064, 2,204 (M), 520 (F), 18–80 y	CS	Self-reported “regular PA (for transport or recreation/leisure purposes)”—yes/no	41.7%	
Abuelmagd et al. [45]	*n* = 90 (F), 32–86 y	CS	Self-reported PA through structured questionnaire interviews	14.4%	
AlOmar et al. [46]	*n* = 100, 33 (M), 67 (F), 20–31 + y	CS	Self-reported questionnaire asking if participants exercise and frequency (few times/month, 1–2 times/week, > 2 times/week, every day)	71%	
DHHS [2]	*n* = 2,533, 4,941 (M), 4,689 (F), 18 + y	CS		19.9%	
Hamoudi et al. [47] phase 1(expatriates)	*n* = 2,406, 1,638 (M), 768 (F), 18 + y	CS	Self-reported PA status	47%	
Hamoudi et al. [47] phase 2/ (emiratis)	*n* = 797, 419 (M), 378 (F), 18 + y	CS	Self-reported PA status (active vs. inactive)	28%	187 excluded for incomplete data
Hamid et al. [6]	*n* = 13,299, 9,629 (M), 4,689 (F), 18 + y	CS		14.9%	
Pramodh [48]	*n* = 493 (F), 18–25 y	CS	Self-reported questionnaire asking frequency of PA per week and types of activities preferred	3.2% performed > 10 h/wk	Students reported that walking (77%) and shopping in malls (42.5%) were their favourite PA
Al Sabbah [49]	*n* = 251 (F), 23 ± 5.3 y	CS	Self-reported questionnaire; exercise ≥ 60 min/day (yes/no)	55.7%	
Hashim et al. [40]	*n* = 202, 50 (M), 152(F)	CS	Self-reported exercise habits via modified Nordic questionnaire (yes/no for regular exercise)	64.9%	
Bawady et al. [50]	*n* = 9,198, 4,743 (M), 4,455 (F), 18–61 y	CS	Self-reported; Regular exercise defined as ≥ 150 min PA per week	7.9%	
Sadeq et al. [51]	*n* = 231, 141 (M), 90 (F)	Retrospective cohort hospital-based study		~ 26.9%	
Gariballa et al. [52]	*n* = 965, 164 (M), 803 (F),	Prospective longitudinal cohort study	Validated questionnaire assessing occupation and leisure PA (frequency/duration of sessions ≥ 20 min causing breathlessness/sweating)	72%	62% obese (BMI ≥ 30), 30% overweight; 26% had diabetes (M), 12% (F); 22% hypertension (M), 13% (F)
AlBlooshi et al. [53]	*n* = 1,537 (F), 18–40 y	CS	Self-reported frequency: Never/rare, 1–2 times/week, 3–5 times/week, daily	Very low: 75.5% inactive/minimal activity (35% never/rare + 40.5% only 1–2 times/week);	31.4% had medical history including diabetes 7.1%, hypertension 5.1%
Mohammad et al. [54]	*n* = 116, 59 (M), 57 (F), 18 + y	CS	Self-reported survey: minutes of PA/wk	39%	
Sharma et al. [55]	*n* = 138, 52 (M), 86 (F), 17–24 y	CS	Self-reported questionnaire asking about lifestyle, diet, exercise; categorized participants as “sedentary”	56.5%	
Smail [56]	*n* = 72 (F), 24–72 y	CS	Self-reported PA (Exercise status: Yes/No)	48.6%	
Mezhal et al. [27]	*n* = 5,058, 3,136 (M), 1,922 (F), 18–40 y	CS	Self-reported questionnaire; classified as moderate-high or low PA	8.8% moderate-high; 37.5% low	53.7% missing data
Abd Alsamad et al. [57]	*n* = 344, 167 (M), 177 (F), 17–25 y	CS	Self-reported questionnaire (WHO STEPS)—frequency, intensity, lifestyle (active vs. sedentary)	65.1%	
Bashier et al. [58]	*n* = 542	CS	Self-reported: minimum 30-min exercise for 5 days/week (per 2021 ESC guidelines)	15%	
Almukhattin et al. [59]	*n* = 200, 58 (M), 142 (F), 19–60 + y	CS	Self-reported classification into 4 levels: Inactive, Low, Medium (150–300 min/week moderate exercise), Highly active (> 300 min/week)	Highly active: 0.5% Medium: 25% Low: 41.5% Inactive: 33%	

*UAE* United Arab Emirates, *CS* cross-sectional survey, *M* male, *F* female, *GPAQ* General Physical Activity Questionnaire, *PA* physical activity, *CDC/ACSM* Centers for Disease Control and Prevention and the American College of Sports Medicine, *BMI* body mass index

**Table 5:** Characteristics of included studies using validated surveys to assess self-reported physical activity or inactivity in the UAE

Study	Sample (no., sex, age)	Design	Physical activity measures and classification criteria	Physical activity prevalence	Notes
Stubbs et al. [60]	*n* = 1,183, 18–69 y	CS	IPAQ	49.1%	UAE had the highest prevalence of low PA among all 38 countries studied
UAE NHS [3]	*n* = 8,214, 4,000 + (M), 4,000 + (F), 18 + y	CS	GPAQ	34%	
Hashim et al. [61]	*n* = 256 (F), 19–40 y	Prospective cohort study	PPAQ	35.2%	Response rate (61%)
Abd-Allatif [62]	*n* = 1,930, 1,375 (M), 555 (F), 18–46 y	CS	IPAQ-SF	24.7%	55 people did not respond
Radwan et al. [63]	*n* = 308, 150 (M), 158 (F), 18–25 y	CS	IPAQ-SF	73.1%	
Shah et al. [64]	*n* = 553 (F), 18–46 + y	CS	IPAQ-SF	53.7%	
Amiri et al. [65]	*n* = 567, 182 (M), 385 (F), 20–65 y	CS	IPAQ-SF	71.4%	
Mohamedali et al. [66]	*n* = 910, 479 (M), 431 (F), 18–50 + y	CS	IPAQ	55.4%	
Radwan et al. [67]	*n* = 448, 221(M), 227 (F), 18–25 y	CS	IPAQ	60.8%	

*UAE* United Arab Emirates, *CS* cross-sectional survey, *M* male, *F* female, *IPAQ* International Physical Activity Questionnaire, *IPAQ-SF* International physical activity questionnaire—short form

**Table 6:** Characteristics of included studies using accelerometers to assess physical activity in the UAE

Study	Sample (number, sex, age)	Design	Physical activity measures and classification criteria	Physical activity prevalence	Notes
Dalibalta et al. [14]	*n* = 139, 62 (M), 77 (F), 17 + y y	CS	ActiGraph wGT3X-BT accelerometer	83.1% (F) and 95.2% (M) met new PA guidelines; only 1.3% (F) and 12.9% (M) met bout-based guidelines	
Qadah et al. [68]	*n* = 156, 18–35 y	CS	Fibion accelerometer	Mean vigorous PA: 4.79 ± 5.85 min/day	Mean sitting: 11.37 ± 1.10 h/16 h day

*UAE* United Arab Emirates, *CS* cross-sectional survey, *M* male, *F* female

### Risk of Bias in Individual Studies

Overall methodological quality varied from low to high, with appraisal scores ranging from 11.1% to 100% and a mean compliance rate of 71.3%. Most studies demonstrated an appropriate sample frame addressing the target population (87.9%), sufficient data coverage of the identified sample (84.5%), adequate sample size (81.0%), and appropriate statistical analysis (77.6%). However, sampling methods (51.7%) and management of response rates (38%) were less consistently reported across studies. Moderate compliance was observed for detailed reporting of subjects and settings (74.1%), while domains related to the validity and reliability of measurement methods, particularly in the identification (56.9%) and standard measurement (56.9%) of conditions, showed lower adherence. More recent studies, particularly those published after 2018, generally demonstrated enhanced methodological rigour, with increased use of validated measurement tools, clearer sampling strategies, and improved reporting practices. Detailed appraisal outcomes for all 60 studies are presented in the OSM (Table S1).

### Results of Syntheses

#### Overall Pooled Estimates of Physical Activity

**Fig. 2:**
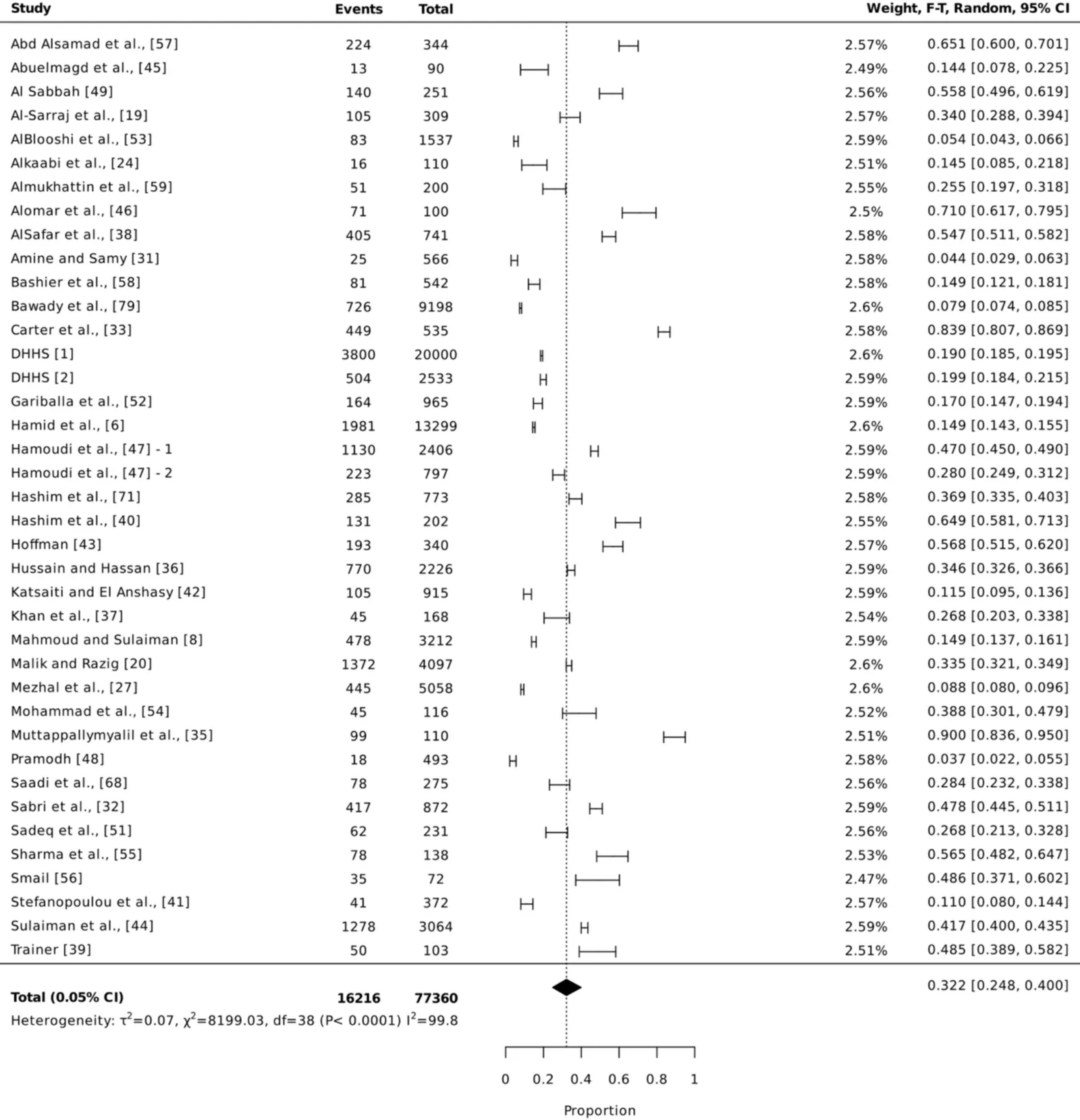
Forest plot of random-effects models for all studies included in the pooled estimation analysis of self-reported PA prevalence. *PA* physical activity, *DHHS* Dubai Household Health Survey, *F-T* Freeman-Tukey

**Fig. 3:**
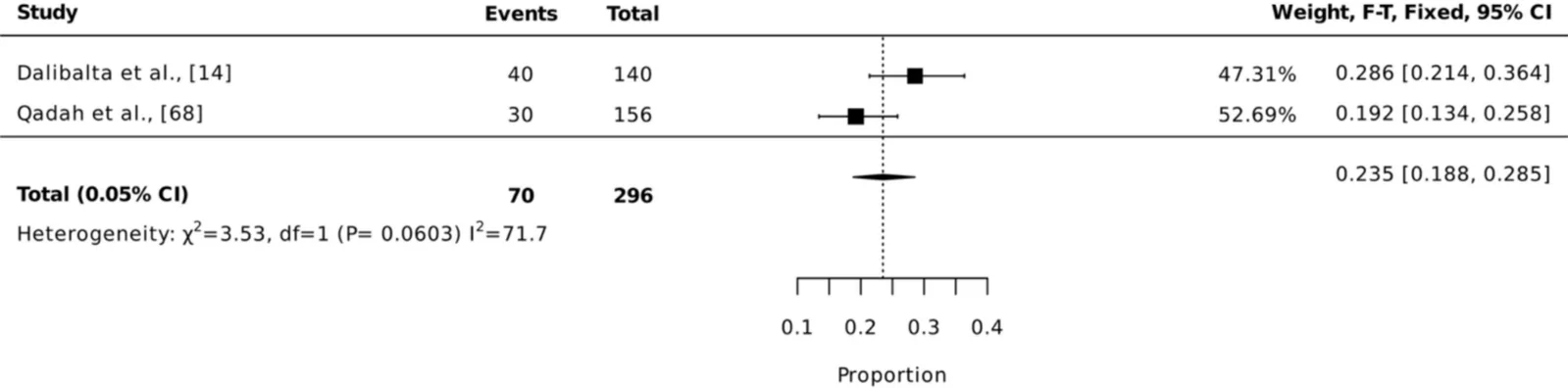
Forest plot of random-effects models for all studies included in the pooled estimation analysis of physical activity prevalence measured using accelerometer-based data. *F-T* Freeman-Tukey

**Fig. 4:**
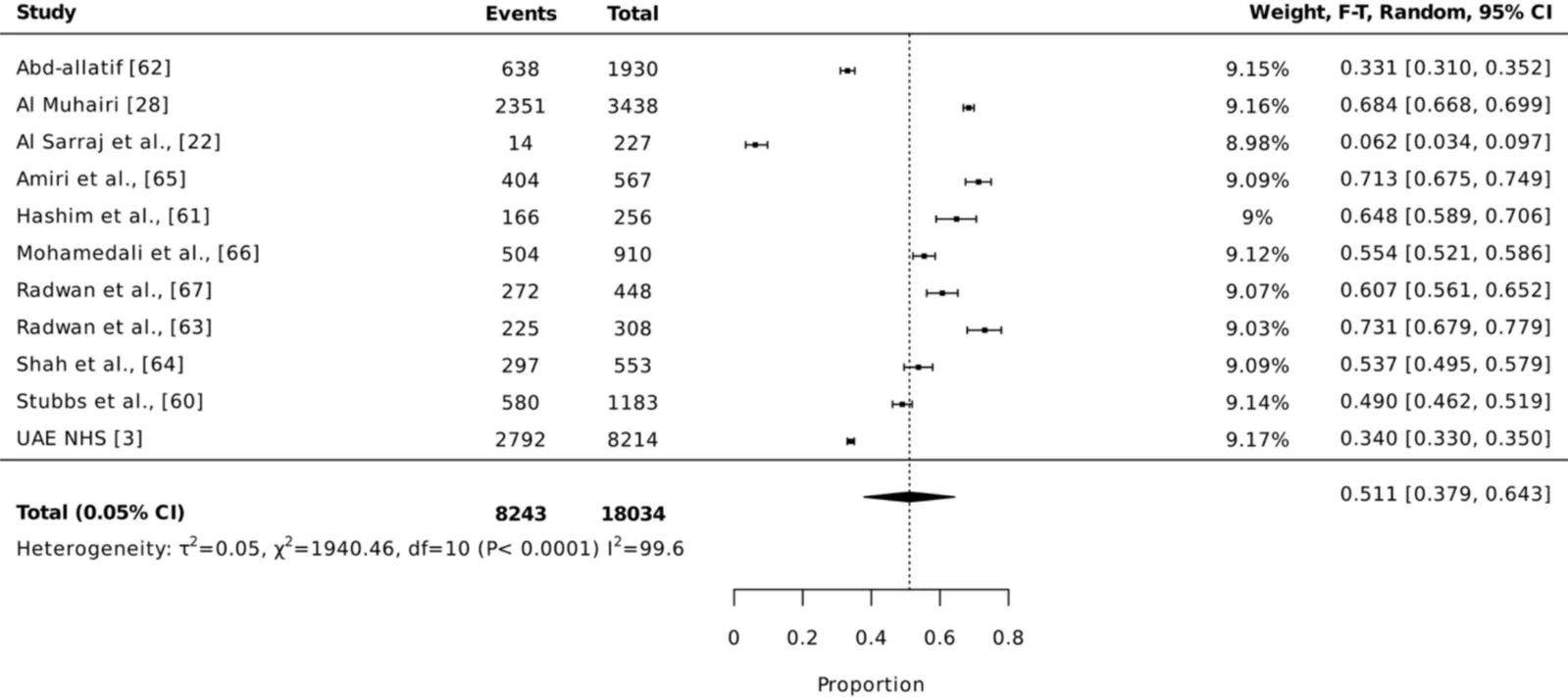
Forest plot of random-effects models for all studies included in the pooled estimation analysis of self-reported physical activity prevalence assessed using validated survey tools. *UAE* United Arab Emirates, *NHS* National Health Survey, *F-T* Freeman-Tukey

A meta-analysis of 39 studies (*n* = 77,360) revealed a pooled PA prevalence of 0.322 (95% CI 0.248–0.4), with substantial heterogeneity (*I*^2^ = 99.8%, *p* < 0.0001) (Fig. 2). The nearly identical study weights observed in the forest plot result from high heterogeneity and the use of a random-effects model, where between-study variance dominates the weighting, as described in the Cochrane Handbook and prevalence meta-analysis literature [69]. In two studies (*n* = 296) utilizing accelerometer-based data, a prevalence of 0.235 (95% CI 0.188–0.285) was observed (Fig. 3). In contrast, the 11 studies (*n* = 18,034) that relied on validated survey instruments for PA reported a pooled prevalence of 0.511 (95% CI 0.379–0.643) (Fig. 4).

#### Prevalence of Physical Inactivity

Objective measurement tools provide the clearest indication of the severity of sedentary behaviour in the UAE. Recent accelerometer-based studies show extremely high levels of sitting time and minimal engagement in vigorous activity. Qadah et al. [68] reported that adults spent an average of 11.37 h per day sitting, with only 4.79 min of vigorous PA. Similarly, Dalibalta et al. [14] found that participants spent approximately 80% of waking hours sedentary (~ 12 + h/day), with MVPA comprising only 4–5.5% of waking time despite high compliance with newer PA guidelines (≥ 150 min/week MVPA, any bout length). These objective data highlight a broader national trend of prolonged sedentary time and limited movement opportunities across UAE populations. Self-reported studies conducted over nearly three decades further reinforce the persistence of physical inactivity as a major public health challenge. The earliest available evidence from Amine and Samy [31] showed that only 4.4% of female university students engaged in any form of structured exercise, establishing an early baseline of widespread inactivity among young Emirati women. Throughout the 2000s, similar patterns were observed: for example, Al-Sarraj et al. [19] found that only 34% of participants had exercised in the 2 weeks preceding data collection. Population-level surveys from the 2010s demonstrated particularly high rates of insufficient activity. The Dubai Household Health Survey (DHHS) [1] showed that only 19% of residents met recommended guidelines. Subsequent studies echoed this trend: Hussain and Hassan [36] found that just 34.6% of Dubai’s adult population regularly engaged in PA, while Alsafar et al. [38] reported that 45.3% of UAE nationals indicated no participation in PA. Hamid et al.’s [6] analysis of subsequent DHHS data revealed that 85.1% of the population were physically inactive, an increase from 2010 and substantially higher than the global prevalence estimate of 17%. More recent national data indicate that inactivity remains widespread. The UAE National Health Survey [3] found that 70.8% of respondents were insufficiently active, with higher rates among Emiratis (80.8%**)** compared to non-Emiratis **(**69.2%). Among clinical populations, inactivity levels are even more concerning. Bawady et al. [50] reported that only 7.9% of individuals with type 2 diabetes exercised regularly, while Sadeq et al. [51] found that 73% of stroke patients were sedentary. Additional studies showed that just 15% of diabetic patients at high cardiovascular risk met PA recommendations [58], and only 49% of cervical cancer patients were physically active [56]. Taken together, both objective and self-reported evidence consistently demonstrate that physical inactivity and sedentary behaviour remain deeply entrenched across the UAE, cutting across demographic groups, nationalities, and health conditions.

#### Overall Pooled Estimates of the Metabolic Syndrome

**Fig. 5:**
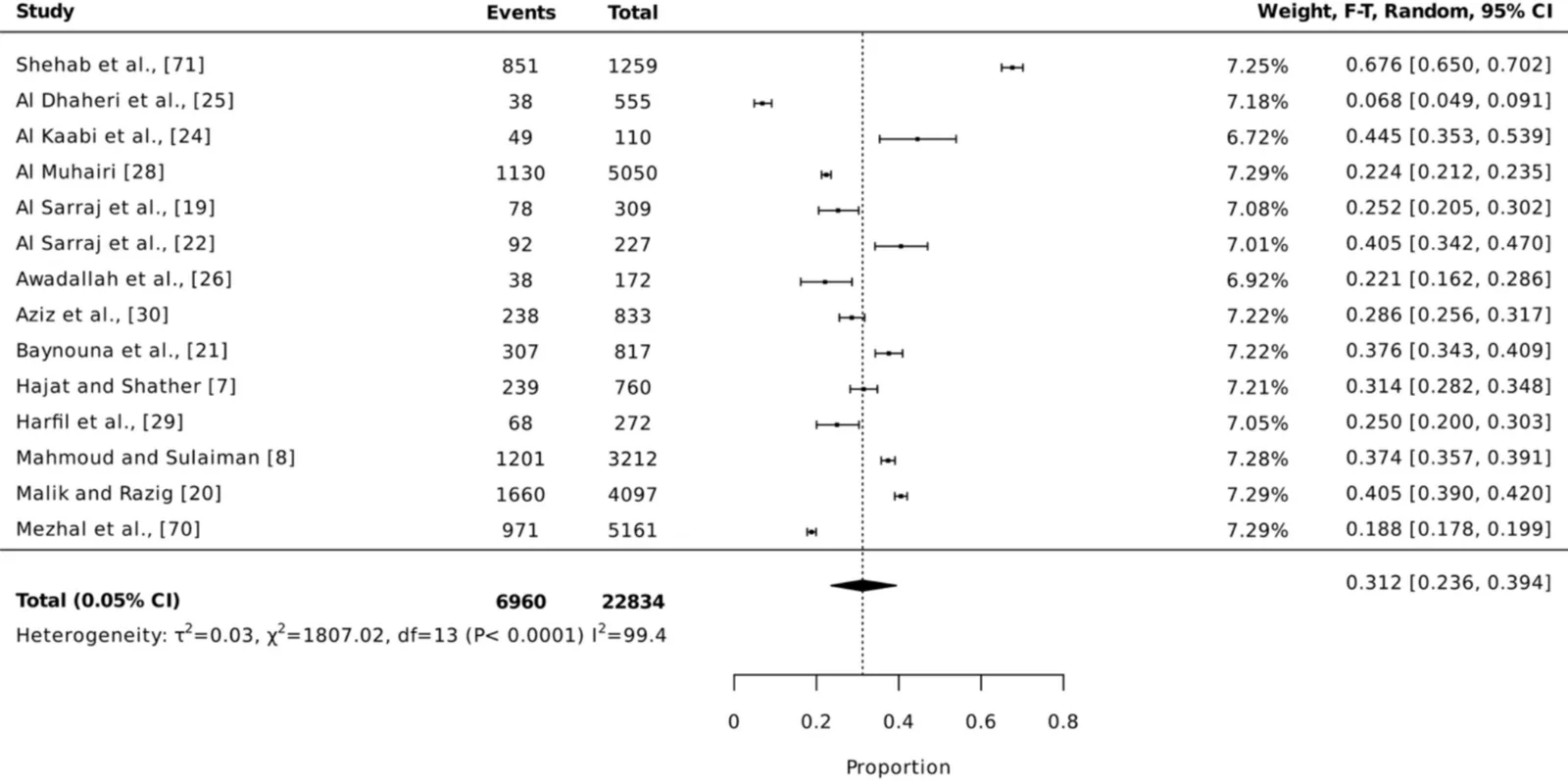
Forest plot of random-effects models for all studies included in the pooled estimation analysis of the metabolic syndrome. *F-T* Freeman-Tukey

Meta-analysis of 14 community-based adult studies (*n* = 22,834) revealed pooled MetS prevalence of 0.312 (95% CI 0.236–0.394), with substantial heterogeneity (*I*^2^ = 98.5%, *p* < 0.001) (Fig. 5). Similarly, in the meta-analysis of MetS, the comparable weights across included studies reflect the high heterogeneity and the statistical approach of the random-effects model, where the between-study variance largely governs the weighting, as supported in methodological literature and the Cochrane Handbook [69].

#### Prevalence of the Metabolic Syndrome

Early comprehensive studies established baseline prevalence. Baynouna et al. [21] reported MetS prevalence of approximately 37.6%, increasing with age and more common in females. Al-Sarraj et al. [19] found 40.5% of overweight/obese Emirati adults met criteria for MetS. Large-scale studies have also confirmed high prevalence. Hajat and Shather [7] reported MetS prevalence of 48.7% (IDF criteria) and 50.3% (ATPIII criteria) in Abu Dhabi, with males showing higher rates. Malik and Razig [20] found approximately 40% of adults had MetS based on both National Cholesterol Education Program (NCEP) (39.6%) and IDF (40.5%) definitions. Studies on specific subgroups revealed varying rates. Alkaabi et al. [24] found 45% of overweight/obese Emirati women met IDF criteria. Among younger populations, Al Dhaheri et al. [25] reported 6.8% prevalence in ages 17–25 years, rising to 34.5% among obese individuals. Recent studies provided updated estimates. Mahmoud and Sulaiman [8] reported 37.4% overall prevalence (men: 39%, women: 32.7%). Mezhal et al. [70] found 22.7% in men and 12.5% in women among young Emirati adults under 40 years of age. Harfil et al. [29] reported 25% prevalence in Sharjah adults, with significantly higher rates in males (36%) than females (18%). AlMuhairi [28] reported 22.4% prevalence among 5,050 young Emirati adults aged 18–40 years. Collectively, these findings demonstrate a persistently high burden of MetS across UAE populations, with prevalence typically ranging between 30 and 50%. Despite methodological and population differences, the evidence indicates that MetS remains a widespread metabolic health concern, particularly among adults with excess body weight and advancing age.

#### Demographic Variations

Demographic variations in PA and MetS across studies in the UAE reveal notable sex, age, and nationality differences. Sex disparities were consistent for PA with females generally less active, for example, the UAE NHS [3] and Mohammad et al. [54] showed females had higher insufficient activity rates (74.8% vs. 66.8%). Radwan et al. [63] found 56.2% of male students were active compared to only 25% of females. Hashim and Al-Ali [71] reported 46% of male dentists exercised regularly versus 28% of females. For MetS, sex-based patterns were complex. Several studies reported higher prevalence in males [7, 29, 70], while others found higher rates in females [20, 21], likely reflecting differences in diagnostic criteria and age distributions. Age emerged as a consistent predictor: PA declined sharply with increasing age, with the DHHS [1] showing only 8.8% of women aged 60 + years were sufficiently active. The UAE NHS [3] identified ages 30–44 years as having the highest insufficient PA. MetS prevalence increased substantially with age across all studies, and Baynouna et al. [21], Mezhal et al. [70], and Mahmoud and Sulaiman [8] all identified age as a significant risk factor. Nationality and ethnicity also played a role. The UAE NHS [3] found Emiratis had notably higher insufficient PA rates (80.8%) versus non-Emiratis (69.2%). Among migrants, Abd-Allatif [62] documented substantial variation: Filipino females showed highest activity (45.9%), while South Asian (10.2%) and Arab females (11.0%) had very low rates. Overall, these demographic trends indicate that physical inactivity and MetS are unequally distributed across UAE populations, with women, older adults, and Emirati nationals consistently exhibiting higher risk profiles. Such disparities highlight the influence of socio-cultural and lifestyle factors that shape health behaviours within the UAE’s diverse population.

#### Temporal Trends

Physical inactivity rates varied substantially depending on measurement criteria. Amine and Samy’s [31] baseline showed 95.6% of female students reporting no regular exercise. Using the stricter standard of meeting recommended exercise guidelines, DHHS data from 2010 and 2019 showed approximately 80–81% not meeting guidelines (with only 19–20% meeting them), suggesting remarkable stability despite public health initiatives. The 2014 Dubai survey (85.1% inactive) and UAE NHS [3] (70.8% insufficient activity) fell within this range, though variations likely reflect methodological differences. Recent objective measurements [14, 68] revealed participants spending 11–12 h daily sitting, confirming deeply entrenched sedentary lifestyles persisted even into the 2020s.

MetS prevalence appeared relatively stable from 2008–2012, clustering around 37–50% depending on criteria and populations. Malik and Razig [20] and Baynouna et al. [21] reported approximately 37–40%, while Hajat and Shather [7] reported 48.7–50.3% in Abu Dhabi. More recent studies from 2022–2024 showed somewhat lower rates in younger populations (Mezhal et al. [70]: 22.7% men, 12.5% women under 40 years of age; AlMuhairi [28]: 22.4% in 18- to 40-year-olds), potentially reflecting generational differences or improved early detection and management rather than true population-level improvement. In summary, inactivity and MetS have shown remarkable persistence across decades, with little evidence of sustained improvement. The consistency of these findings points to enduring behavioural and environmental influences that continue to shape population health in the UAE.

#### Physical Activity Assessment

Substantial heterogeneity existed in PA measurement. Early studies used simple self-report questions about any exercise [19, 31]. Mid-period studies (2010–2018) employed validated questionnaires like IPAQ [60], while recent studies (post-2019) increasingly used objective tools [14, 68]. The activPAL accelerometer was employed by Dalibalta et al. [14] and Qadah et al. [68], providing detailed objective data on sitting, standing, and activity time. Large surveys [1–3] used standardized questionnaires aligned with WHO recommendations. Definitions of “sufficient” PA varied: some used ≥ 150 min/week of moderate activity, others used ≥ 30 min daily walking, and still others categorized PA by intensity levels (vigorous/moderate/light). This methodological heterogeneity substantially complicated cross-study comparisons and temporal trend assessment. The diversity of measurement approaches highlights both progress and challenge. While the transition toward validated and objective instruments marks an important methodological advance, inconsistent definitions of “sufficient activity” have hampered clear temporal and cross-study comparisons.

#### Metabolic Syndrome Diagnostic Criteria

Studies employed multiple diagnostic criteria including ATP III (modified and original), IDF, NCEP, and harmonized criteria. Key differences included waist circumference thresholds (European vs. ethnic-specific), triglyceride cutoffs, and whether central obesity was mandatory (IDF) or three of five components (ATP III). Component prevalence varied substantially across studies. Hajat and Shather [7] reported central obesity as most prevalent (66.6%), followed by high triglycerides (39.6%), low high-density lipoprotein cholesterol (HDL-C) (39.3%), high blood pressure (38.6%), and elevated fasting glucose (34.9%). Mahmoud and Sulaiman [8] similarly found waist circumference most common (57.2%), followed by blood pressure (48%), reduced HDL-C (38.3%), triglycerides (35%), and fasting glucose (30%). However, lipid profiles in Emiratis revealed distinctive patterns. Al-Sarraj et al. [19] found only 7% had elevated triglycerides despite 40.5% meeting MetS criteria, with 95% having elevated low-density lipoprotein cholesterol (LDL-C). Similarly, Alkaabi et al. [24] reported low triglyceride prevalence (10%) among overweight/obese women, though 82% had dyslipidaemia characterized predominantly by low HDL-C (80%) and 94% had a large waist circumference. This unique pattern—high LDL-C, low HDL-C, but low triglycerides—contradicted typical MetS presentations and suggested possible genetic factors unique to this population, rendering triglycerides “essentially irrelevant” as a diagnostic criterion in this group. In combination, these findings underscore both the progress and challenges in understanding metabolic health within the UAE. While surveillance has become more sophisticated and awareness has grown, varying diagnostic interpretations and population-specific lipid patterns highlight the need for harmonized methodologies and context-specific prevention strategies moving forward.

#### Sensitivity Analyses

Leave-one-out sensitivity analyses were conducted to assess the robustness of the pooled prevalence estimates by sequentially excluding each contributing study and recalculating the pooled effect [18]. For MetS, excluding each of the 14 included studies in turn yielded pooled estimates ranging from 28.5% (95% CI 22.5–34.9) to 33.6% (95% CI 26.6–40.9), with the original pooled estimate of 31.2% (95% CI 23.6–39.4) falling near the centre of this range. For PA assessed using validated survey instruments (IPAQ/GPAQ), the pooled prevalence of physical inactivity ranged from 48.8% (95% CI 35.2–62.6) to 56.4% (95% CI 47.2–65.3) across leave-one-out iterations, compared with the original estimate of 51.1% (95% CI 37.9–64.3). For PA assessed using other self-report methods, the pooled prevalence ranged from 30.6% (95% CI 23.8–37.9) to 33.2% (95% CI 25.9–41.0), with the original estimate of 32.2% (95% CI 24.8–40) sitting near the upper end of this range. For PA assessed using accelerometry, a leave-one-out analysis was not feasible due to the small number of contributing studies (*n* = 2); the pooled estimate of 23.5% (95% CI 18.8–28.5) from these two studies should therefore be interpreted with caution, and further research using objective measurement is needed to confirm the finding. Across the three meta-analyses where leave-one-out analysis was feasible, the 95% CIs overlapped between iterations and no single study shifted any pooled estimate more than approximately five percentage points, supporting the robustness of the overall prevalence findings.

### Reporting Biases

Formal assessment of small-study or publication bias using Egger’s regression test or funnel plot inspection was not performed, as these methods have limited validity when applied to meta-analyses of prevalence proportions and may produce misleading results in this context. Instead, reporting bias was considered narratively. The search strategy was designed to minimise reporting bias through systematic searches of eight peer-reviewed databases combined with comprehensive grey literature sources, including thesis repositories, conference proceedings, and government health surveillance reports (UAE Ministry of Health and Prevention, Dubai Health Authority, Abu Dhabi Department of Health), supplemented by hand-searching of reference lists from included studies. No date restrictions were applied at the search stage. Notwithstanding these measures, the inclusion of only English-language publications and the possibility that some institutional reports and unpublished surveillance datasets may not have been retrievable means that residual reporting bias cannot be fully excluded. Overall, the risk of reporting bias across the four syntheses was judged to be low to moderate.

### Certainty of Evidence

The certainty of the pooled prevalence estimates was appraised narratively across the four GRADE [72] domains most applicable to prevalence syntheses: risk of bias, inconsistency, indirectness, and imprecision. Risk of bias was rated as moderate, given that included studies had a mean JBI compliance of 71.3% but with notable limitations in sampling methods (51.7%) and management of response rates (38%). Inconsistency was rated as substantial for all four pooled estimates, reflecting the very high heterogeneity observed (*I*^2^ = 99.8% for physical inactivity and *I*^2^ = 98.5% for MetS) arising from methodological, measurement, and population diversity across studies. Indirectness was rated as low, as included studies addressed the target population (UAE adults) and outcomes of interest (physical inactivity and MetS prevalence) directly. Imprecision was rated as low for the overall pooled prevalence estimates, given the large, pooled sample sizes (*n* = 77,360 for physical activity and *n* = 22,835 for MetS) and the robustness of the estimates demonstrated by the leave-one-out sensitivity analyses. On balance, the overall certainty of evidence for the pooled prevalence of physical inactivity and MetS in the UAE was judged to be low to moderate, with inconsistency driven by heterogeneity in measurement and diagnostic criteria being the principal factor downgrading certainty.

## Discussion

The findings highlight a sustained and escalating physical inactivity burden in the UAE. Although systematic surveillance began only in the mid-1990s, low activity levels likely predate formal evidence. Early studies revealed near-universal inactivity among Emirati women [31] and predominantly sedentary lifestyles among adults [19]. Despite repeated public-health recommendations, national surveys across the 2010s continued to show 70–85% of adults as insufficiently active, with inactivity consistently higher among Emiratis and women [3, 6]. Recent accelerometer-based studies confirm these trends, showing Emiratis spend 11–12 h daily sitting and perform almost no vigorous activity [14, 68]. Although not included in this review due to methodological scope, accelerometer studies in the UAE by Alsamman et al. [73, 74] objectively measured 10–11 h daily sitting time among Emirati women, highlighting that self-reported prevalence data likely underestimate the true extent of inactivity. Together, these data demonstrate that inactivity has persisted across decades and populations, necessitating a deeper exploration of the structural, environmental, and cultural factors that sustain this pattern.

Multiple structural and environmental determinants underpin this trend. Rapid urbanization has produced car-centric city designs with limited pedestrian infrastructure [66]. Trainer [39] similarly reported that only two surveyed students engaged in walking for errands, while all others relied exclusively on motor vehicles. The UAE’s accelerated socio-economic transformation compressed lifestyle changes that elsewhere unfolded over a century into just a few decades, magnifying the population’s transition toward physical inactivity. The extreme climate, with summer temperatures exceeding 40 °C for months, has created additional barriers. Combined with wealth sufficient to make air-conditioned indoor living universal, this effectively eliminates the necessity for outdoor activity during much of the year. Few major urban centres globally combine such extreme heat with infrastructure that makes avoiding outdoor exposure entirely feasible. In addition, cultural factors have also played a huge role in this problem, with women facing persistent restrictions. Alkaabi et al. [24] noted cultural barriers and lack of facilities for women, while Stefanopoulou et al. [41] identified socio-cultural norms restricting outdoor activities and lack of culturally sensitive exercise facilities. The magnitude of sex disparity in the UAE (74.8% female inactivity vs. 66.8% male inactivity) reflects barriers that persisted from Amine and Samy’s [31] observations through to the most recent surveys. Specifically, work-related time constraints emerge as the most cited barrier. Hussain and Hassan [36] found 47.3% cited lack of time, a finding consistent across different occupational groups. For instance, Hashim and Al-Ali [71] reported 43% of dentists also gave this reason for not prioritizing PA, and also cited work schedule demands as preventing them from prioritizing PA. The UAE NHS [3] found that only 26.8% identified “PA time during work hours” as something that would encourage more activity, suggesting work structures may be fundamentally incompatible with active lifestyles. The progression from awareness to action remains elusive. Hussain and Hassan [36] documented a striking gap, whereby 86.6% showed positive attitudes toward PA, yet only 34.6% actually practised it. Khan et al. [37] found 79% recognized exercise benefits for diabetes prevention, but only 27% exercised regularly. Studies using validated instruments found only 51% achieved adequate PA levels, though this likely overestimated activity given response bias, as seen in AlMuhairi [28] where only 68.4% (3,438/5,050) of participants providing metabolic markers completed PA questions. This awareness-practice gap persists despite decades of health promotion, suggesting that knowledge alone is insufficient without addressing structural, cultural, and environmental barriers.

### Metabolic Syndrome: High Prevalence in a Regional and Global Context

The pooled prevalence of MetS of 31.2% is comparable to rates in other developed nations. However, when considering metabolic health outcomes, particularly MetS, the burden appears considerably higher. MetS prevalence shows relative stability over time, though at levels substantially elevated compared to international benchmarks. From 2008–2012, prevalence clustered around 37–50% depending on criteria and populations, with such rates being considerably higher than those typically observed globally. While global MetS prevalence ranges from 20 to 25% in developed nations and 10% to 15% in developing nations, the UAE’s rates position it among regions with elevated metabolic burden. The most striking finding is Hajat and Shather’s [7] report that nearly half (48.7–50.3%) of the sample from Abu Dhabi met criteria for MetS. This was not limited to older adults or clinical populations but represented the general adult population. Malik and Razig [20] confirmed similar patterns nationwide with 40% prevalence across both NCEP and IDF definitions. For context, the USA reports MetS prevalence of approximately 34%, while European nations typically range from 20 to 30%, highlighting that the UAE faces a substantially elevated burden.

Age patterns are consistent and concerning. Baynouna et al. [21] noted prevalence increasing with age, yet even younger populations showed worrying rates. Al Dhaheri et al. [25] found 6.8% of 17- to 25-year-olds had MetS, and while this is seemingly low in absolute terms, this represents metabolic dysfunction in individuals who should be in peak health. More alarmingly, the prevalence increased to 34.5% among obese young adults. In most developed nations, MetS prevalence below age 40 years typically remains under 10%, suggesting the UAE population experiences metabolic dysfunction at younger ages than international norms. The 2020s data suggest possible shifts in younger populations. Mezhal et al. [70] reported 22.7% prevalence in men and 12.5% in women under 40 years of age, while AlMuhairi [28] found 22.4% in 18- to 40-year-olds. These rates, while still concerning and elevated compared to international benchmarks for this age group, are lower than the 37–50% seen in general adult populations, potentially reflecting either cohort effects, improved early detection and management, or true differences between younger and older Emiratis.

The most scientifically significant finding is the documentation of a unique Emirati lipid pattern by Al-Sarraj et al. [19] and Alkaabi et al. [24]. Despite 40.5% meeting MetS criteria, only 7% had elevated triglycerides, while 95% exhibited elevated LDL-C—a pattern distinct from the typical MetS. This “distinctive pattern of high LDL-C, low HDL-C, and low triglycerides” contrasts with the classic “atherogenic dyslipidaemia” seen worldwide, which features elevated triglycerides, low HDL-C, and increased small dense LDL particles with usually normal or mildly elevated LDL-C. The Emirati lipid profile essentially inverts this relationship, suggesting possible genetic polymorphisms unique to this population or influences from lifestyle factors such as prolonged sedentary behaviour. Notably, prolonged sitting and physical inactivity are known to adversely affect HDL-C, glucose metabolism, and blood pressure without necessarily elevating triglycerides, which may help explain this lipid pattern [75]. These findings highlight the need for population-specific cardiometabolic risk assessments beyond traditional lipid markers. The authors noted this rendered triglyceride “essentially irrelevant as a diagnostic criterion for MetS in this group, unlike in most other populations worldwide.” This finding has profound implications: standard international diagnostic criteria, developed primarily in Western populations and validated in Asian and Hispanic populations, appear to potentially misclassify metabolic risk in Emiratis. While population-specific adaptations have been recognized for certain ethnic groups, particularly Asians requiring lower waist circumference thresholds, this unique lipid pattern suggests the need for more fundamental reconsideration of diagnostic approaches in Gulf Arab populations.

Component prevalence reveals central obesity as the dominant feature. Hajat and Shather [7] found 66.6% had central obesity, the highest of all components. Alkaabi et al. [24] reported 94% of overweight/obese women exceeded waist circumference thresholds. This aligns with international patterns where central adiposity typically shows highest prevalence, though the near-universal presence (94%) among overweight/obese individuals exceeds rates in most populations globally, where even among the obese, typically 70–80% exceed waist circumference thresholds. Awadallah et al.’s [26] finding that waist circumference was “the strongest independent predictor” of oxidative stress (β = 0.45, *p* < 0.001) provides mechanistic insight. The combination of near-universal central obesity among overweight/obese individuals with the extremely low PA documented earlier, suggests a direct link: sedentary behaviour promotes abdominal fat accumulation, which drives metabolic dysfunction through inflammatory and oxidative stress pathways. Sex-based patterns are complex and criterion dependent. Several studies [7, 29, 70] found higher male prevalence, while others [20, 21] reported higher female prevalence. Component analysis reveals the source: women have higher central obesity and lower HDL-C, while men have higher blood pressure and triglycerides [8, 29]. Thus, depending on whether criteria emphasize central obesity (IDF) or equally weighted five components (ATP III), sex-specific prevalence patterns shift. This variability, while present internationally, appears more pronounced in the UAE context. Physiologically, these sex-based patterns reflect differential responses to low PA that converge on the MetS pathway. In women, inactivity reduces muscle oxidative capacity and HDL synthesis while promoting visceral fat accumulation and inflammatory cytokine release; in men, it heightens sympathetic drive, hepatic triglyceride production, and vascular dysfunction. Despite differing mechanisms, both trajectories culminate in systemic insulin resistance, the core pathophysiological process underlying MetS [11].

### The Physical Inactivity-Metabolic Syndrome Association

The temporal stability of MetS (despite persistently high physical inactivity), reflects MetS' multifactorial nature. While physical inactivity clearly contributes, dietary patterns, genetic factors, and rapid urbanization all play roles. Several studies directly examined this relationship. AlMuhairi [28] provided the strongest evidence: physically inactive individuals had 34% higher odds of MetS (adjusted odds ratio (OR) = 1.34), with leisure-time PA particularly protective against MetS and related metabolic abnormalities. The association was strongest for abdominal obesity and elevated triglycerides, components most directly influenced by energy balance. This effect size (OR = 1.34) aligns with international meta-analyses, which typically report ORs of 1.3–1.5 for the physical inactivity-MetS association. However, the population-level impact differs substantially from international contexts. While the individual-level effect is similar, the population attributable risk is much higher in the UAE because of the extreme prevalence of inactivity. When 80% of a population is inactive (compared to 30–40% in many developed nations), even with similar ORs, approximately twice as many MetS cases are attributable to inactivity. This underscores the urgency of population-wide interventions.

Some studies found non-significant associations in multivariate models. Malik and Razig [20] reported PA “not significantly associated with MetS in the multivariate analysis” despite being significant univariately, while Hamoudi et al. [47] found similar patterns (OR = 1.09, *p* = 0.403). This likely reflects complex confounding: physical inactivity associated with obesity, which strongly predicts MetS, making independent effects difficult to isolate statistically. Moreover, physically inactive individuals tend to have multiple concurrent risk factors (poor diet, smoking, low health literacy) that together drive MetS. The Al-Sarraj et al. [19, 22] studies illuminate this complexity. Extremely low PA (only 6% minimally active) co-existed with 40.5% MetS prevalence, yet the unique lipid pattern (normal triglycerides, high LDL-C) differed from typical sedentary populations elsewhere. This suggests genetic factors moderate how physical inactivity manifests metabolically in Emiratis, with atherogenic lipid profiles developing even without the usual triglyceride elevation seen in other inactive populations worldwide, such as those in Spain [76], Saudi Arabia [77], China [78], and the USA [79], where physical inactivity is commonly associated with elevated triglycerides, low HDL-C, and atherogenic lipid profiles. Clinical populations reveal downstream consequences. Shehab et al. [23] found that among acute coronary syndrome patients, hyperglycaemia and low HDL-C predicted mortality, while MetS itself did not, suggesting individual components matter more than the syndrome per se, for clinical outcomes. Sadeq et al. [51] identified low PA as the third strongest predictor of ischemic stroke (OR = 4.2). These findings underscore that MetS represents not merely statistical clustering but genuine cardiovascular risk requiring urgent intervention.

### Methodological Considerations and Their Implications

The substantial methodological heterogeneity across studies complicates temporal and cross-population comparisons, potentially obscuring true trends. For PA, definitions range from “any exercise in the past 2 weeks” [19] to ≥ 150 min per week of moderate activity (WHO standard) to objective accelerometry [68]. Studies using liberal definitions (any activity) report lower inactivity prevalence than those using strict criteria (150 min per week), potentially creating artefactual temporal patterns when methodological rigor increased over time. The shift toward objective measurement post-2019 represents important methodological progress. Accelerometry reveals that individuals self-reporting as “active” often accumulate insufficient moderate-to-vigorous activity when objectively measured [14]. This “social desirability bias” means earlier self-report studies likely under-estimated true inactivity prevalence, suggesting the historical situation may have been worse than recognized.

For MetS, criterion choice substantially affects prevalence estimates and their clinical meaning. Hajat and Shather [7] found IDF criteria more strongly associated with diabetes (relative risk (RR) = 6.4) but ATPIII better predicted cardiovascular disease (RR = 13.6). The mandating of central obesity in IDF criteria means populations with high obesity prevalence show higher MetS prevalence under IDF versus ATPIII definitions. Waist circumference thresholds are particularly challenging. Hajat and Shather [7] noted that using European thresholds may overestimate prevalence in Middle Eastern populations, yet ethnic-specific thresholds remain debated. Given that 94% of overweight/obese Emirati women exceeded even European waist circumference thresholds [24], this debate has limited practical impact—central obesity is nearly universal among those with elevated BMI (body mass index) regardless of cut point. The unique Emirati lipid pattern has profound diagnostic implications. Standard international criteria emphasizing triglycerides as one of five components appear to miss metabolic risk in populations where triglycerides remain normal despite other metabolic derangements. This suggests the need for population-specific diagnostic approaches or alternative lipid markers (apolipoprotein B, LDL particle number) that might better capture cardiovascular risk. The lack of standardized measurement protocols hampers rigorous trend assessment. Without consistent methodology across time, apparent changes could reflect measurement rather than genuine epidemiological shifts. Future surveillance requires standardized, validated, culturally appropriate tools applied consistently to enable meaningful temporal comparisons.

### Clinical and Public Health Implications

The combination of physical inactivity with MetS prevalence represents a substantial public health challenge requiring comprehensive intervention. While health education initiatives have increased awareness, the persistence of high inactivity rates suggests that complementary structural and environmental interventions are needed to translate awareness into sustained behavioural change. Structural and environmental modifications are essential. Urban planning requires fundamental re-thinking. Cities need pedestrian-friendly infrastructure with shaded walkways, abundant parks, and connected walking/cycling networks. Climate-controlled indoor facilities are essential given extreme summer heat. Women’s-only facilities addressing cultural concerns are necessary but insufficient; thus, broader cultural shifts enabling female outdoor activity are equally important. International examples demonstrate feasibility: cities like Copenhagen transformed from car-dependent to bicycle-friendly over several decades through sustained infrastructure investment and policy prioritization.

Workplace interventions show promise. Hashim and Al-Ali [71] found private sector dentists exercised more than public sector counterparts due to flexible split shifts versus continuous 8-h blocks. The UAE NHS [3] found only 26.8% identified workplace activity time as encouraging, suggesting most workplaces do not facilitate PA. Structured activity breaks, on-site facilities, and activity-promoting policies (standing desks, walking meetings) could leverage workplaces as intervention settings, as demonstrated in Scandinavian countries where workplace PA programs achieved substantial participation. However, replicating such models in the UAE must account for environmental constraints, as prolonged outdoor activity is often unfeasible during extreme heat, necessitating climate-adapted indoor or shaded interventions.

For MetS, the unique Emirati lipid pattern demands reconsideration of diagnostic approaches. Standard international criteria might miss metabolic risk in populations with the Al-Sarraj et al. [19] pattern. Ethnic-specific criteria or alternative lipid markers might better capture cardiovascular risk. This requires further research but had immediate clinical implications for risk stratification and treatment decisions.

**Fig. 6:**
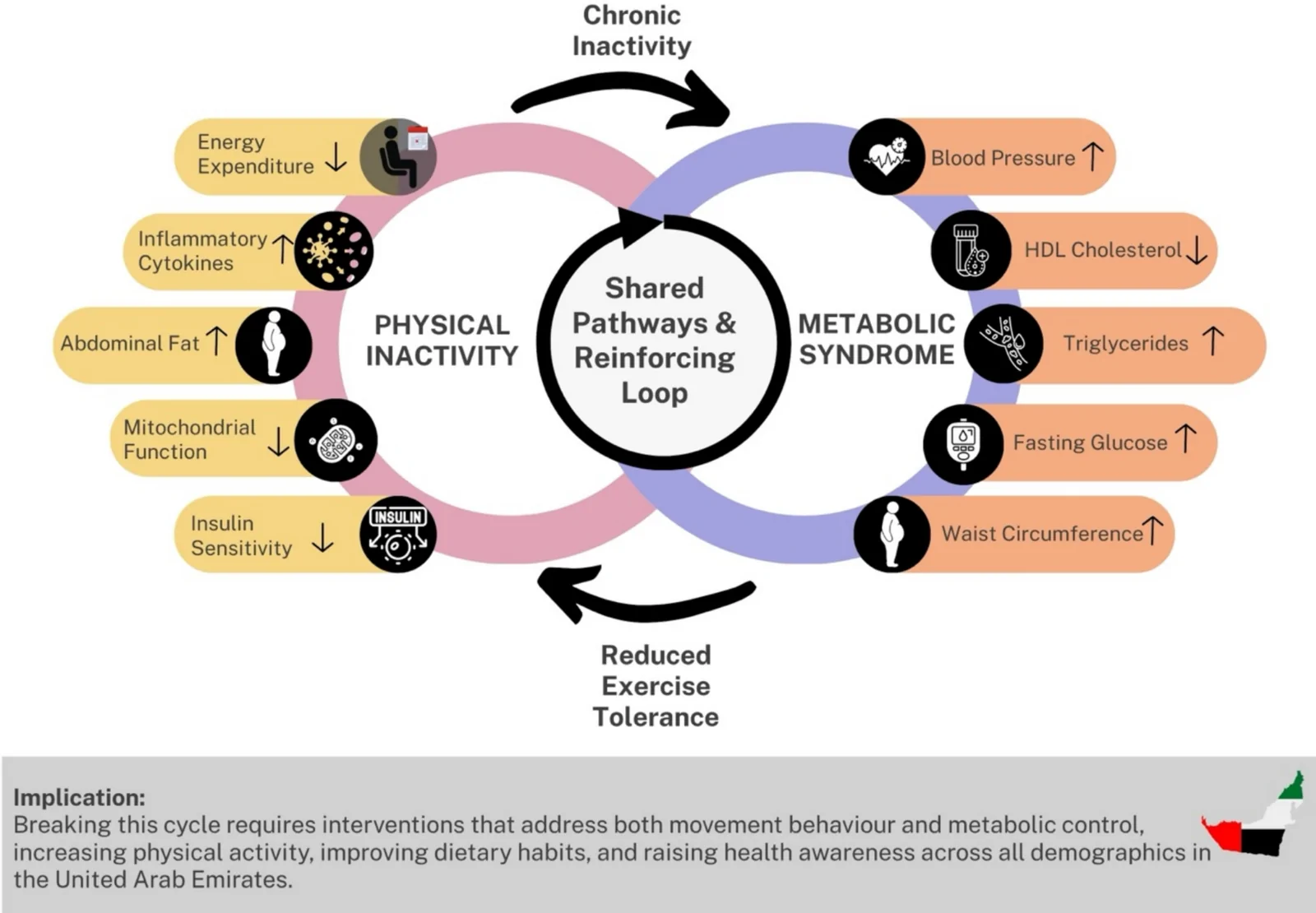
Cycle showing how physical inactivity and MetS reinforce each other through shared pathways. *MetS* metabolic syndrome, *HDL* high-density lipoprotein

The finding that individual MetS components predicted outcomes better than the syndrome per se [23] suggests component-specific interventions might prove more effective than syndrome-focused approaches. Targeting hypertension, hyperglycaemia, and dyslipidaemia individually based on mechanistic understanding could yield better clinical outcomes than treating MetS as a unified entity. Yet this component-focused approach must be reconciled with the reality that physical inactivity functions as a shared upstream driver affecting all MetS components simultaneously. The relationship between physical inactivity and MetS is bidirectional and self-reinforcing (Fig. 6). Chronic inactivity promotes MetS through decreased energy expenditure, increased inflammation, abdominal fat accumulation, mitochondrial dysfunction, and reduced insulin sensitivity [75]. Conversely, MetS components (elevated blood pressure, dyslipidaemia, hyperglycaemia, central obesity) reduce exercise tolerance, creating barriers to PA [80]. In the UAE, where 67.2% of adults are physically inactive and 31.2% have MetS, this reinforcing cycle has become entrenched at the population level.

## Limitations

Several limitations should be acknowledged when interpreting these findings. First, substantial heterogeneity was observed across the included studies (*I*^2^ = 99.8% for physical inactivity, *I*^2^ = 98.5% for MetS), reflecting diverse methodologies, measurement instruments, diagnostic criteria, and target populations and complicating direct comparisons. Second, the majority of included studies employed cross-sectional designs, precluding causal inference about how physical inactivity drives MetS development over time. Third, objective measurement of physical activity through accelerometry was limited to only two studies, restricting confidence in the objectively measured pooled estimate. Fourth, included studies varied in JBI methodological quality scores (range 11.1–100%), with notable variability in sampling methods (51.7% compliance) and response rate management (38%), which may have introduced selection bias into individual prevalence estimates. Finally, the review was restricted to English-language publications and may have missed relevant Arabic-language reports, though this limitation is partially offset by the extensive grey literature search including UAE government surveillance reports.

## Recommendations for Future Research

The limitations identified above point to several priorities for future research. Addressing these gaps will be essential to strengthen the evidence base, refine intervention strategies, and inform population-level policy in the UAE.

Given the UAE’s hierarchical governance structure, future research should explore how governmental leadership can be leveraged to strengthen national-level strategies promoting PA and metabolic health. Understanding how 'top-down' policy initiatives can translate into community engagement and sustained behavioural change is critical. At a more operational level, healthcare professionals, particularly doctors and medical practitioners, hold a uniquely trusted position within Emirati society. Investigating how their influence can be harnessed alongside fitness professionals may enhance community-level dissemination of evidence-based health messages.

Methodologically, future research should employ standardized, validated measurement tools applied consistently across populations and time periods. Objective assessment of PA through accelerometry should become standard in surveillance to reduce self-report bias and enable accurate trend evaluation. MetS assessment should also consider ethnic-specific criteria and emerging biomarkers that reflect population diversity within the UAE. Longitudinal cohort studies following individuals over time would clarify temporal relationships between physical inactivity and MetS development, identify critical intervention windows, and quantify how activity changes influence metabolic outcomes.

Greater attention to subgroup-specific patterns is also needed. Sex differences in physical inactivity (74.8% in females vs. 66.8% in males), ethnic variation across UAE residents, and age-specific trajectories, particularly the elevated MetS prevalence (22.4–22.7%) observed among adults under 40 years of age, each warrant dedicated investigation. Research disaggregating these subgroups would support more targeted intervention design and more equitable resource allocation.

Intervention research is urgently needed. Randomized controlled trials testing environmental modifications, workplace-based programmes, culturally appropriate women’s initiatives, and policy interventions could identify effective strategies within the UAE context. Given that international models may not directly translate across cultural or climatic settings, locally developed and tested approaches are essential. Implementation science examining how to scale successful pilot programmes would facilitate translation from efficacy to population-level effectiveness. Qualitative studies exploring why awareness–practice gaps persist could further inform culturally sensitive designs, identifying resilience factors and positive deviance patterns that sustain activity despite barriers.

## Conclusion

Despite significant government investment in health awareness campaigns and infrastructure development over three decades, the UAE faces a persistent physical inactivity challenge, with 33% meeting activity guidelines compared to 69% globally. Inactivity trends show limited improvement from 95.6% (1996) to current extreme sedentary behaviour (11–12 h daily sitting), despite national initiatives. Combined with elevated MetS prevalence (31.2%), physical inactivity increases MetS odds by 34%, creating substantial population-attributable cardiovascular risk. Critical disparities persist: Emiratis show higher inactivity (80.8%) than non-Emiratis (69.2%), and females (74.8%) exceed males (66.8%). While education-based approaches have raised awareness, evidence suggests the need for complementary structural interventions to achieve sustained behavioural change. Building upon existing government health initiatives, enhanced multisectoral action is recommended: climate-adapted pedestrian infrastructure; sex-specific programs addressing cultural barriers; workplace activity policies; and expanded MetS screening. The convergence of elevated sedentariness, metabolic disease prevalence, and demographic disparities presents an opportunity for evidence-based intervention tailored to UAE’s unique climate, rapid urbanization, and cultural context to mitigate cardiovascular burden.

## Supplementary Information

Below is the link to the electronic supplementary material.

Supplementary file1 (PDF 173 KB)

Supplementary file2 (PDF 261 KB)

Supplementary file3 (DOCX 41 KB)

## Declarations

### Data availability statement

All data generated or analysed during this systematic review and meta-analysis are included in this published article and its supplementary information files. The source data for individual studies are available in the original publications cited in the reference list.
