# Supplementary material for: Physical Inactivity and Metabolic Syndrome in the United Arab Emirates: A Systematic Review and Meta-analysis of Parallel Epidemics
Source: Sports Med. 2026 Jul 22;56(12):3075–98. doi: 10.1007/s40279-026-02493-6 (PMC13759644; doi:10.1007/s40279-026-02493-6)
Supplement: Supplementary file 1 [file 40279_2026_2493_MOESM1_ESM.pdf]

Search Strategy: Physical Inactivity and Metabolic Syndrome in the UAE

Search Strategy Overview

A comprehensive, three-step search strategy will be employed to identify both published and unpublished studies relevant to physical inactivity and metabolic syndrome in the UAE.

Database Search Strategies

PubMed/MEDLINE

```
(("Physical Inactivity"[Mesh] OR "Sedentary Behavior"[Mesh] OR "Motor
Activity"[Mesh] OR physical inactivity[tiab] OR sedentary[tiab] OR
sedentary behaviour[tiab] OR sedentary behavior[tiab] OR low physical
activity[tiab] OR insufficient activity[tiab] OR physical activity[tiab] OR
exercise[tiab])

AND

("Metabolic Syndrome"[Mesh] OR metabolic syndrome[tiab] OR metabolic
syndrome X[tiab] OR insulin resistance[tiab] OR syndrome X[tiab] OR
dysmetabolic syndrome[tiab])

AND

("United Arab Emirates" OR "United Arab Emirates"[tiab] OR UAE[tiab] OR Abu
Dhabi[tiab] OR Dubai[tiab] OR Sharjah[tiab] OR Ajman[tiab] OR "Umm Al
Quwain"[tiab] OR "Ras Al Khaimah"[tiab] OR Fujairah[tiab]))
```

Filters applied: up to June 2025

Embase (Ovid)

```
('physical activity':ab,ti OR 'physical inactivity':ab,ti OR 'insufficient
physical activity':ab,ti OR 'sedentary behavior':ab,ti OR 'sedentary
behaviour':ab,ti OR 'exercise':ab,ti) AND ('metabolic syndrome':ab,ti OR
'metabolic syndrome X':ab,ti OR 'insulin resistance syndrome':ab,ti OR
'syndrome X':ab,ti OR 'dysmetabolic syndrome':ab,ti) AND ('united arab
emirates':ab,ti OR 'uae':ab,ti OR 'emirates':ab,ti OR 'abu dhabi':ab,ti OR
'dubai':ab,ti OR 'sharjah':ab,ti OR 'ajman':ab,ti OR 'umm al quwain':ab,ti
OR 'ras al khaimah':ab,ti OR 'fujairah':ab,ti) AND [english]/lim AND [up to
2025]/py
```

CINAHL (EBSCOhost)

```
Search #1: ("physical activity"[TI,AB] OR "physical inactivity"[TI,AB] OR
"insufficient physical activity"[TI,AB] OR "sedentary behavior"[TI,AB] OR
"sedentary behaviour"[TI,AB] OR "exercise"[TI,AB])
```

```
Search #2: ("metabolic syndrome"[TI,AB] OR "metabolic syndrome X"[TI,AB] OR
"insulin resistance syndrome"[TI,AB] OR "syndrome X"[TI,AB] OR
"dysmetabolic syndrome"[TI,AB])

Search #3: ("United Arab Emirates"[TI,AB] OR "UAE"[TI,AB] OR
"Emirates"[TI,AB] OR "Abu Dhabi"[TI,AB] OR "Dubai"[TI,AB] OR
"Sharjah"[TI,AB] OR "Ajman"[TI,AB] OR "Umm Al Quwain"[TI,AB] OR "Ras Al
Khaimah"[TI,AB] OR "Fujairah"[TI,AB])

Search #4: (#1 AND #2 AND #3)
```

Filters: English language, research articles, up to June 2025

Scopus

```
TITLE-ABS-KEY("physical activity") OR TITLE-ABS-KEY("physical inactivity")
OR TITLE-ABS-KEY("insufficient physical activity") OR TITLE-ABS-
KEY("sedentary behavior") OR TITLE-ABS-KEY("sedentary behaviour") OR TITLE-
ABS-KEY("exercise") AND TITLE-ABS-KEY("metabolic syndrome") OR TITLE-ABS-
KEY("metabolic syndrome X") OR TITLE-ABS-KEY("insulin resistance syndrome")
OR TITLE-ABS-KEY("syndrome X") OR TITLE-ABS-KEY("dysmetabolic syndrome")
AND TITLE-ABS-KEY("United Arab Emirates") OR TITLE-ABS-KEY("UAE") OR TITLE-
ABS-KEY("Emirates") OR TITLE-ABS-KEY("Abu Dhabi") OR TITLE-ABS-KEY("Dubai")
OR TITLE-ABS-KEY("Sharjah") OR TITLE-ABS-KEY("Ajman") OR TITLE-ABS-KEY("Umm
Al Quwain") OR TITLE-ABS-KEY("Ras Al Khaimah") OR TITLE-ABS-KEY("Fujairah")
AND PUBYEAR < 2026 AND (LIMIT-TO(DOCTYPE,"ar")) AND (LIMIT-
TO(LANGUAGE,"English"))
```

Web of Science

```
("physical activity" OR "physical inactivity" OR "insufficient physical
activity" OR "sedentary behavio*" OR "exercise") AND ("metabolic syndrome"
OR "metabolic syndrome X" OR "insulin resistance syndrome" OR "syndrome X"
OR "dysmetabolic syndrome") AND ("United Arab Emirates" OR "UAE" OR
"Emirates" OR "Abu Dhabi" OR "Dubai" OR "Sharjah" OR "Ajman" OR "Umm Al
Quwain" OR "Ras Al Khaimah" OR "Fujairah")
```

Filters: English language, articles, up to June 2025

PsycINFO (EBSCOhost)

```
Search #1: ("physical activity" OR "physical inactivity" OR "insufficient
physical activity" OR "sedentary behavior" OR "sedentary behaviour" OR
"exercise").ti,ab.

Search #2: ("metabolic syndrome" OR "metabolic syndrome X" OR "insulin
resistance syndrome" OR "syndrome X" OR "dysmetabolic syndrome").ti,ab.

Search #3: ("United Arab Emirates" OR "UAE" OR "Emirates" OR "Abu Dhabi" OR
"Dubai" OR "Sharjah" OR "Ajman" OR "Umm Al Quwain" OR "Ras Al Khaimah" OR
"Fujairah").ti,ab.

Search #4: (#1 AND #2 AND #3)
```

Filters: English language, up to June 2025

Cochrane Library

```
("physical activity" OR "physical inactivity" OR "insufficient physical
activity" OR "sedentary behavior" OR "sedentary behaviour" OR "exercise")
AND ("metabolic syndrome" OR "metabolic syndrome X" OR "insulin resistance
syndrome" OR "syndrome X" OR "dysmetabolic syndrome") AND ("United Arab
Emirates" OR "UAE" OR "Emirates" OR "Abu Dhabi" OR "Dubai" OR "Sharjah" OR
"Ajman" OR "Umm Al Quwain" OR "Ras Al Khaimah" OR "Fujairah")
```

Filters: Up to June 2025

SPORTDiscus (EBSCOhost)

```
Search #1: ("physical activity" OR "physical inactivity" OR "insufficient
physical activity" OR "sedentary behavior" OR "sedentary behaviour" OR
"exercise").ti,ab.

Search #2: ("metabolic syndrome" OR "metabolic syndrome X" OR "insulin
resistance syndrome" OR "syndrome X" OR "dysmetabolic syndrome").ti,ab.

Search #3: ("United Arab Emirates" OR "UAE" OR "Emirates" OR "Abu Dhabi" OR
"Dubai" OR "Sharjah" OR "Ajman" OR "Umm Al Quwain" OR "Ras Al Khaimah" OR
"Fujairah").ti,ab.

Search #4: (#1 AND #2 AND #3)
```

Filters: English language, up to June 2025

Grey Literature and Additional Sources

ProQuest Dissertations & Theses

```
ab("physical activity" OR "physical inactivity" OR "sedentary behavior" OR
"sedentary behaviour" OR "exercise") AND ab("metabolic syndrome" OR
"insulin resistance syndrome" OR "syndrome X") AND ab("United Arab
Emirates" OR "UAE" OR "Emirates" OR "Abu Dhabi" OR "Dubai")
```

OpenGrey

```
("physical activity" OR "physical inactivity" OR "sedentary behavior" OR
"exercise") AND ("metabolic syndrome" OR "insulin resistance syndrome") AND
("United Arab Emirates" OR "UAE" OR "Emirates")
```

Google Scholar

With all of the words: physical inactivity metabolic syndrome UAE **With the exact phrase:** "physical inactivity" "metabolic syndrome" **With at least one of the words:** "United Arab Emirates" UAE Emirates "Abu Dhabi" Dubai Sharjah Ajman "Umm Al Quwain" "Ras Al Khaimah" Fujairah

Regional and Specialized Sources

- **UAE Health Research Portal**
- **UAE Ministry of Health and Prevention reports**
- **Dubai Health Authority publications**

- **Abu Dhabi Department of Health reports**
- **Conference proceedings:** International Conference on Public Health in the Arab World, Gulf Cooperation Council Health Ministers' meetings
- **WHO Regional Office for the Eastern Mediterranean (EMRO) reports**
- **Arab Board of Health Specializations publications**

Search Strategy Implementation Steps

Step 1: Initial Limited Search

- Conduct preliminary search in PubMed and CINAHL
- Analyze text words from titles, abstracts, and index terms
- Develop comprehensive search strategy in consultation with librarian

Step 2: Comprehensive Database Search

- Execute search strategy across all eight electronic databases
- Search grey literature sources
- Include English-language studies up to December 2025
- Include non-English studies where translation is feasible

Step 3: Reference and Citation Screening

- Manual screening of reference lists from included articles
- Forward citation tracking of relevant reviews
- Google and Google Scholar searches for additional grey literature

Search Terms Legend

- **TI:** Title
- **AB:** Abstract
- **TW:** Text word
- **MeSH:** Medical Subject Headings
- **ab,ti:** Abstract and title fields (Embase format)

Quality Assurance

- Search strategy developed and reviewed by subject librarian
- Pilot testing of search terms across databases
- Documentation of all search modifications and rationale
- Regular consultation with research team throughout search process
