# Supplementary material for: Physical Inactivity and Metabolic Syndrome in the United Arab Emirates: A Systematic Review and Meta-analysis of Parallel Epidemics
Source: Sports Med. 2026 Jul 22;56(12):3075–98. doi: 10.1007/s40279-026-02493-6 (PMC13759644; doi:10.1007/s40279-026-02493-6)
Supplement: Supplementary file 2 [file 40279_2026_2493_MOESM2_ESM.pdf]

NIHR | National Institute for Health and Care Research

PROSPERO
International prospective register of systematic reviews

Physical Inactivity and Metabolic Syndrome in the UAE: Parallel Epidemics? A Systematic Review and Meta-analysis

Bushra Ali, Chris Bishop, Tim Evans, Anne Elliott

Citation 1 change

Bushra Ali, Chris Bishop, Tim Evans, Anne Elliott. Physical Inactivity and Metabolic Syndrome in the UAE: Parallel Epidemics? A Systematic Review and Meta-analysis. PROSPERO 2025 CRD420251065730. Available from https://www.crd.york.ac.uk/PROSPERO/view/CRD420251065730.

REVIEW TITLE AND BASIC DETAILS

Review title 1 change

Physical Inactivity and Metabolic Syndrome in the UAE: Parallel Epidemics? A Systematic Review and Meta-analysis

Condition or domain being studied 1 change

Metabolic Syndrome ; Physical activity

Rationale for the review 1 change

Physical inactivity is a major modifiable risk factor for MetS, a cluster of conditions that significantly increases the risk of cardiovascular disease and diabetes. In the UAE, rapid economic growth and urbanization have led to substantial lifestyle changes, resulting in high rates of physical inactivity and sedentary behavior, particularly among youth and women. Recent studies indicate that nearly 58% of UAE adults are physically inactive, with a corresponding high prevalence of overweight and obesity—key components of MetS.

Despite the recognized link between physical inactivity and MetS, there is limited region-specific synthesis of the available evidence, especially for the UAE, where cultural, environmental, and demographic factors may influence these associations. A systematic review is needed to comprehensively assess the prevalence and relationship between physical inactivity and MetS in the UAE, identify research gaps, and inform targeted public health interventions. This review will add to the existing knowledge by providing an up-to-date, UAE-specific synthesis of observational evidence, highlighting unique regional patterns, and supporting the development of tailored strategies to reduce the burden of metabolic syndrome in this rapidly changing society.

The UAE presents a unique opportunity to examine physical inactivity and metabolic syndrome as potentially parallel epidemics. Understanding the prevalence patterns of both conditions, how they vary across different population segments, and how they have evolved over time is essential for evidence-based public health planning. This review adopts a prevalence-focused approach to provide a comprehensive epidemiological profile of both conditions in the UAE context, which will inform resource allocation, screening programs, and prevention strategies.

Review objectives 1 change

Review Objectives:

To systematically identify and synthesize all available evidence on the prevalence of physical inactivity and metabolic syndrome among adults in the UAE

To examine how prevalence rates of both conditions vary across demographic subgroups (age, gender, nationality) and geographic regions within the UAE

To assess temporal trends in the prevalence of physical inactivity and metabolic syndrome in the UAE

To evaluate the measurement approaches and diagnostic criteria used to assess physical inactivity and metabolic syndrome in UAE studies

To identify gaps in the current literature and make recommendations for future research and public health interventions

Primary Question:

What is the current evidence on the prevalence of physical inactivity and metabolic syndrome in UAE populations?

Secondary Questions:

Demographic Variations: How do the prevalence rates vary across demographic subgroups (age, gender, nationality) and geographic regions within the UAE?

Temporal Question: How have the prevalence rates of physical inactivity and metabolic syndrome changed over time in the UAE?

Methodological Question: What measurement approaches and diagnostic criteria have been used to assess physical inactivity and metabolic syndrome in UAE studies?

Keywords

Metabolic syndrome; Physical activity; UAE

Country

United Arab Emirates

ELIGIBILITY CRITERIA

Population 1 change

Included

Physical Inactivity: Studies must report prevalence data on physical inactivity or sedentary behavior among UAE adults. Physical activity/inactivity may be measured using any of the following methods:

Quantitative measures:

• Validated questionnaires (e.g., IPAQ, GPAQ)

• Objective measures (e.g., accelerometers, pedometers, heart rate monitors)

• Quantified physical activity levels (e.g., minutes per week, MET-minutes, step counts)

• Defined as not meeting established guidelines

Binary/categorical measures:

• Self-reported dichotomous physical activity status ("active" vs. "inactive" or "sedentary")

• Single-item questions

• Categorical classifications

• Studies using any definition or cut-off for classifying individuals as physically active or inactive

All measurement methods will be included regardless of validation status, with quality and comparability assessed during risk of bias evaluation and addressed in sensitivity analyses.

Metabolic Syndrome: Studies must report prevalence data on metabolic syndrome defined according to established criteria such as:

• ATP III

• IDF

• WHO

• Harmonized criteria

Studies reporting on both conditions or either condition independently are eligible.

Adults aged 18 years and older who are residents (including both citizens and expatriates) of the UAE, regardless of gender, nationality, ethnicity, or socioeconomic status.

Studies including both Emirati nationals and non-nationals living in any of the seven Emirates will be considered.

No restrictions on language, setting (urban or rural), or population subgroup (e.g., general population, working adults, university students), as long as participants are residents of the UAE

Excluded
• Studies that exclusively evaluate intervention effects without reporting baseline prevalence data

• Studies focusing solely on other health conditions without assessing physical inactivity or metabolic syndrome prevalence

Intervention(s) or exposure(s)

Included
Physical activity; Participation In Leisure Activity

Inclusion Criteria

Physical Inactivity and Sedentary Behavior:

Studies must assess physical inactivity or sedentary behavior as a primary exposure. Physical inactivity should be defined as not meeting established physical activity guidelines (e.g., less

than 150 minutes of moderate-intensity activity per week), and may be measured by self-report questionnaires, accelerometers, pedometers, or validated tools.

Measurement Methods:

Both subjective (e.g., International Physical Activity Questionnaire, self-administered surveys) and objective (e.g., accelerometers, pedometers) measures of physical inactivity or sedentary time are eligible.

Study Designs:

Observational studies (cross-sectional, cohort, case-control) that report on the relationship between physical inactivity or sedentary behavior and metabolic syndrome or its components among UAE residents. Qualitative studies (e.g., interviews, focus groups, ethnographic studies)

Population:

Adults (≥18 years) residing in the UAE, regardless of gender, nationality, or socioeconomic status.

Exclusion Criteria

Intervention-Only Studies:

Studies that exclusively evaluate the effects of interventions to increase physical activity (e.g., structured exercise programs, workplace interventions) without reporting baseline or habitual physical inactivity levels as an exposure or without examining the association between inactivity and metabolic syndrome.

Non-Physical Activity Exposures:

Studies focusing solely on other lifestyle factors (e.g., diet, smoking, pharmacological interventions) without assessing physical inactivity or sedentary behavior as an exposure.

Comparator(s) or control(s)

This review does not have any comparators

Study design

Both randomized and nonrandomized study types will be included.

Context

The UAE is experiencing a rapid rise in non-communicable diseases, with MetS emerging as a major public health concern, particularly among both Emirati nationals and expatriate populations. This trend is closely linked to lifestyle changes driven by urbanization and economic growth, which have contributed to high rates of physical inactivity and sedentary behavior across the country. Research from the broader MENA region, including the UAE, consistently shows that a significant proportion of adults and youth do not meet recommended physical activity guidelines, with females and younger people especially affected.

Physical inactivity and sedentary behaviors, such as prolonged screen time, have been identified as significant risk factors for the development of metabolic syndrome and its components in both adolescents and adults. However, the measurement and reporting of physical inactivity and sedentary behavior vary widely, and there is a lack of standardized guidelines for their quantification in the region. Despite these challenges, evidence indicates that addressing physical inactivity should be a public health priority in the UAE, as it is strongly

associated with increased MetS prevalence and related risk factors such as obesity and diabetes.

This review is conducted in the context of a high and growing burden of metabolic syndrome in the UAE, with the aim of synthesizing all available evidence on the relationship between physical inactivity and MetS among UAE residents. By providing a comprehensive and up-to-date summary of observational data, this review will help identify key risk groups, inform targeted public health interventions, and highlight gaps in the current research landscape.

TIMELINE OF THE REVIEW

Date of first submission to PROSPERO
13 June 2025

Review timeline
Start date: 2 June 2025. End date: 1 January 2026.

Date of registration in PROSPERO
16 June 2025

AVAILABILITY OF FULL PROTOCOL

Availability of full protocol
A full protocol has been written and uploaded to PROSPERO. The protocol will be made available after the review is completed.

SEARCHING AND SCREENING

Search for unpublished studies
Both published and unpublished studies will be sought.

Main bibliographic databases that will be searched
The main databases to be searched are *CINAHL - Cumulative Index to Nursing and Allied Health Literature*, *Embase - Embase via Ovid*, *Embase.com*, *PsycInfo*, *PubMed* and *Scopus*.

Other important or specialist databases that will be searched
Web of Science (broad multidisciplinary coverage)

Cochrane Library (systematic reviews and trials; for context and related evidence)

SPORTDiscus

Search language restrictions
There are no language restrictions.

Search date restrictions
There are no search date restrictions.

Other methods of identifying studies
No other methods will be used.

Link to search strategy

A full search strategy has been uploaded to PROSPERO. The PDF may be accessed through this link https://www.crd.york.ac.uk/PROSPEROFILES/192ae0cd89ba15a2b242524330a9b958.pdf.

Selection process

Studies will be screened independently by at least two people (or person/machine combination) with a process to resolve differences.

Other relevant information about searching and screening

None

DATA COLLECTION PROCESS

Data extraction from published articles and reports

Data will be extracted independently by at least two people (or person/machine combination) with a process to resolve differences.

Authors will be asked to provide any required data not available in published reports.

Study risk of bias or quality assessment 1 change

Risk of bias will be assessed using:

Risk of bias and methodological quality will be assessed using the appropriate JBI Critical Appraisal Tools:

• JBI Critical Appraisal Checklist for Studies Reporting Prevalence Data

• JBI Critical Appraisal Checklist for Analytical Cross-Sectional Studies (if applicable)

• JBI Critical Appraisal Checklist for Cohort Studies (for longitudinal prevalence data)

Data will be assessed independently by at least two people (or person/machine combination) with a process to resolve differences.

Additional information will **not** be sought from study investigators if required information is unclear or unavailable in the study publications/reports.

Reporting bias assessment

Risk of bias due to missing results will be assessed

Certainty assessment 1 change

The certainty of evidence will be assessed using the GRADE approach adapted for prevalence studies. The assessment will consider:

• Risk of Bias: Quality of included studies using JBI critical appraisal results

• Inconsistency: Heterogeneity in prevalence estimates (I^2 statistic)

• Indirectness: Applicability to UAE adult population

• Imprecision: Width of confidence intervals and sample sizes

• Publication Bias: Assessment via funnel plots and Egger's test

For prevalence outcomes, certainty will be rated as high, moderate, low, or very low. Two reviewers will independently assess certainty, with disagreements resolved through discussion

or third-reviewer consultation.

Note: Given the nature of prevalence studies (observational designs), evidence typically starts at "low certainty" and may be upgraded based on factors such as large effect sizes, dose-response gradients (if applicable), or large magnitude of effect.

Summary of findings tables will be prepared using GRADEpro software, presenting:

• Pooled prevalence estimates with 95% confidence intervals

• Number of studies and participants

• Certainty of evidence ratings

• Key findings for each outcome

OUTCOMES TO BE ANALYSED

Main outcomes 1 change

Primary Outcomes:

1. Prevalence of Metabolic Syndrome:

o Point prevalence of metabolic syndrome in UAE adult populations

o Defined according to established criteria (ATP III, IDF, WHO, or Harmonized definitions)

o Including measurements of: waist circumference, blood pressure, fasting glucose, triglycerides, and HDL cholesterol

2. Prevalence of Physical Inactivity:

o Point prevalence of physical inactivity in UAE adult populations

o Assessed using validated tools (IPAQ, GPAQ) or objective measures (accelerometers, pedometers)

o Including proportion not meeting recommended physical activity guidelines

Secondary Outcomes:

1. Demographic Stratification:

o Prevalence estimates stratified by:

 Age groups

 Gender

 Nationality (Emirati vs. non-Emirati)

 Socioeconomic status (if reported)

2. Geographic Variation:

o Prevalence estimates by Emirates or urban/rural settings

3. Temporal Trends:

o Changes in prevalence over time (where multiple time-point data available)

4. Components of Metabolic Syndrome:

o Prevalence of individual MetS components:

 Central obesity (elevated waist circumference)

 Hypertension (elevated blood pressure)

 Hyperglycemia (elevated fasting glucose)

 Hypertriglyceridemia (elevated triglycerides)

 Low HDL cholesterol

5. Measurement Characteristics:

o Types of diagnostic criteria used for MetS

o Types of physical activity assessment tools used

o Specific definitions and cut-offs for physical inactivity:

 Quantitative thresholds (e.g., <150 min/week)

 Binary classifications and their definitions

 Validated vs. non-validated single-item measures

o Quality and reliability of measurement methods

6. Association Between Physical Inactivity and Metabolic Syndrome (Exploratory):

o Where available, odds ratios (OR), risk ratios (RR), or prevalence ratios comparing physically inactive to active individuals

o Stratified prevalence estimates (MetS prevalence by activity status)

o 95% confidence intervals for all effect measures

o Adjusted and unadjusted estimates (where available)

Note: Given the anticipated limited availability of UAE-specific studies directly examining associations, this outcome will be analyzed descriptively if data are insufficient for meta-analysis. Where direct association data are lacking, indirect evidence will be examined through:

o Temporal correlation of prevalence trends

o Overlapping demographic patterns

o Associations with individual MetS components

Additional outcomes

There are no additional outcomes.

PLANNED DATA SYNTHESIS

Strategy for data synthesis 1 change

Data synthesis will be conducted using JBI SUMARI (System for the Unified Management, Assessment and Review of Information) software, following JBI methodology for systematic reviews of prevalence and incidence data.

Quantitative Synthesis: Where studies are sufficiently homogeneous, prevalence data will be pooled using meta-analysis:

• Prevalence meta-analysis will use a random-effects model with double arcsine transformation (Freeman-Tukey) or logit transformation to stabilize variances

• Separate meta-analyses will be conducted for:

o Overall prevalence of metabolic syndrome

o Overall prevalence of physical inactivity

o Subgroup analyses by demographic characteristics (age, gender, nationality)

o Subgroup analyses by geographic region

o Temporal trends (by study period/year)

• Heterogeneity will be assessed using I^2 statistic and Cochran's Q test

• Forest plots will be generated for all meta-analyses

• Funnel plots and Egger's test will be used to assess publication bias where ≥10 studies are available

Sensitivity Analyses:

• By study quality (high vs. low quality studies)

• By MetS diagnostic criteria used

• By physical activity assessment method:

o Validated questionnaires (IPAQ, GPAQ) vs. objective measures vs. simple self-report

o Quantitative measures vs. binary classification

o Objective vs. all subjective measures

• By population type (general population vs. specific subgroups)

• By physical activity definition/cut-off used

Analysis of Associations (if data permit): If sufficient studies (≥3) report association measures or stratified prevalence data:

• Pool effect estimates (ORs, RRs, or prevalence ratios) using random-effects meta-analysis

• If only stratified prevalence data available, calculate prevalence ratios and pool these

• Conduct subgroup analysis by adjustment for confounders

If insufficient data for quantitative synthesis of associations:

• Provide descriptive summary of available association data

• Examine indirect evidence through:

o Temporal correlation analysis (correlation between physical inactivity and MetS prevalence trends over time)

o Demographic pattern comparison (identify if conditions share similar demographic risk profiles)

o Component-level analysis (associations between physical activity and individual MetS components)

• Contextualize UAE findings within international evidence base

Narrative Synthesis: Where meta-analysis is not appropriate due to substantial heterogeneity or insufficient data, a structured narrative synthesis will be conducted:

• Summarizing prevalence estimates in tables

• Describing patterns across demographic groups and time periods

• Discussing methodological variations and their potential impact

• Identifying gaps in the evidence base

Temporal Trend Analysis: If sufficient longitudinal or repeated cross-sectional data are available, temporal trends will be analyzed descriptively or using meta-regression where appropriate.

CURRENT REVIEW STAGE

Stage of the review at this submission 1 change

Review stage	Started	Completed
Pilot work	✓	
Formal searching/study identification	✓	✓
Screening search results against inclusion criteria	✓	✓
Data extraction or receipt of IPD	✓	
Risk of bias/quality assessment		
Data synthesis		

Review status

The review is currently planned or ongoing.

Publication of review results 1 change

Results of the review will be published in English.

REVIEW AFFILIATION, FUNDING AND PEER REVIEW

Review team members 1 change

Mrs Bushra Ali (review guarantor and contact) Middlesex university london. England.

No conflict of interest declared.

Dr Chris Bishop. Middlesex University London. England.

No conflict of interest declared.

Professor Tim Evans. Middlesex university london. England.

No conflict of interest declared.

Dr Anne Elliott. Middlesex university london. England.

No conflict of interest declared.

Named contact

Mrs Bushra Ali (sa3222@live.mdx.ac.uk). Middlesex university london. England.

Review affiliation

Middlesex University London

Funding source

Review has no funding and no agreed support from an academic institution and is done in authors' own time.

Peer review

There has been no peer review of this planned review.

ADDITIONAL INFORMATION

Review conflict of interest

Declared individual interests are recorded under team member details.. No additional interests are recorded for this review.

Medical Subject Headings

Metabolic Syndrome; Exercise; Prevalence; Risk Factors; United Arab Emirates; Adult; Cross-Sectional Studies; Cohort Studies; Case-Control Studies; Obesity; Obesity, Abdominal; Hypertension; Dyslipidemias; Glucose Intolerance

Revision note 1 change

This amendment reflects a refinement of the review's research focus from examining associations between physical inactivity and metabolic syndrome to documenting the parallel prevalence of both conditions in the UAE population. The methodology has been updated to align with JBI (Joanna Briggs Institute) systematic review standards for prevalence studies.

SIMILAR REVIEWS

Check for similar records already in PROSPERO

PROSPERO identified a number of existing PROSPERO records that were similar to this one (last check made on 2 June 2025). These are shown below along with the reasons given by that the review team for the reviews being different and/or proceeding.

- Metabolic syndrome and physical activity in Sub-Saharan Africa: A systematic review and meta-analysis [published 11 June 2024] [CRD42024552281]. The review was judged **not to be similar**
- Physical activity and the risk of cardiovascular disease in adults with metabolic syndrome: a systematic review and dose-response meta-analysis [published 6 September 2023] [CRD42023457891]. The review was judged **not to be similar**
- Prevalence of Physical Inactivity and Sedentary Behavior in Latin American University Students: A systematic review [published 27 April 2022] [CRD42022321342]. The review was judged **not to be similar**
- The relationship of physical activity and health indicators in children and adolescents from 4 to 17 years - an umbrella review [published 9 July 2020] [CRD42020191049]. The review was judged **not to be similar**
- Interventions for sedentary behaviour and physical inactivity to prevent or manage sarcopenia for older adults in nursing homes: a protocol of a systematic review and logic model [published 14 February 2023] [CRD42023394385]. The review was judged **not to be similar**

- The Effect of Physical Inactivity on Cardiovascular Inflammatory Markers in the Geriatric Population in Africa [published 12 June 2024] [CRD42024553602]. The review was judged **not to be similar**
- Systematic review and meta-analysis on population attributable fraction for physical inactivity to dementia under different definitions: are we missing the shot? [published 27 November 2022] [CRD42022375169]. The review was judged **not to be similar**
- The economic burden of physical inactivity: a systematic review and appraisal of methodologies [published 14 September 2016] [CRD42016047705]. The review was judged **not to be similar**
- Individualization and effectiveness of mobile health interventions to reduce physical inactivity and sedentary behavior in healthy children and adolescents: A systematic review and meta-analysis [published 19 November 2020] [CRD42020209417]. The review was judged **not to be similar**
- Free-living physical behaviours and sarcopenia and its determinants in older adults: systematic review and meta-analysis of observational studies [published 9 November 2022] [CRD42022315865]. The review was judged **not to be similar**
- The Correlation Between Daily Physical Activity Levels and ADHD Symptom Severity in Children and Adolescents: A Systematic Review of Step Count and Unstructured Activity Interventions [published 10 October 2024] [CRD42024595703]. The review was judged **not to be similar**

PROSPERO version history 1 change

- Version 2.0, published 07 Oct 2025
- Version 1.0, published 16 Jun 2025

Disclaimer

The content of this record displays the information provided by the review team. PROSPERO does not peer review registration records or endorse their content.

PROSPERO accepts and posts the information provided in good faith; responsibility for record content rests with the review team. The guarantor for this record has affirmed that the information provided is truthful and that they understand that deliberate provision of inaccurate information may be construed as scientific misconduct.

PROSPERO does not accept any liability for the content provided in this record or for its use. Readers use the information provided in this record at their own risk.

Any enquiries about the record should be referred to the named review contact
