# Supplementary material for: Physical Inactivity and Metabolic Syndrome in the United Arab Emirates: A Systematic Review and Meta-analysis of Parallel Epidemics
Source: Sports Med. 2026 Jul 22;56(12):3075–98. doi: 10.1007/s40279-026-02493-6 (PMC13759644; doi:10.1007/s40279-026-02493-6)
Supplement: Supplementary file 3 [file 40279_2026_2493_MOESM3_ESM.docx]

**Table S1.** Summary of quality appraisal of included studies using JBI critical appraisal checklist

| **Citation** | **Q1** Was the sample frame appropriate to address the target population? | **Q2** Were study participants sampled in an appropriate way? | **Q3** Was the sample size adequate? | **Q4** Were the subjects and the setting described in detail? | | **Q5** Was the data analysis conducted with sufficient coverage of the identified sample? | **Q6** Were valid methods used for the identification of the condition? | **Q7** Was the condition measured in a standard, reliable way for all participants? | **Q8** Was there appropriate statistical analysis? | **Q9** Was the response rate adequate, and if not, was the low response rate managed appropriately? | **%** | |  |
| --- | --- | --- | --- | --- | --- | --- | --- | --- | --- | --- | --- | --- | --- |
| **Metabolic Syndrome Prevalence** | | | | | | | | | | | |  | |
| Al-Sarraj et al., [19] | Y | U | Y | | U | Y | Y | Y | Y | N | 66.7 | |  |
| Baynouna et al., [21] | Y | U | Y | | U | U | Y | U | Y | N | 44.4 | |  |
| Al-Sarraj et al., [22] | Y | U | Y | | Y | Y | Y | Y | Y | N | 77.8 | |  |
| Shehab et al., [23] | N | Y | Y | | Y | Y | Y | Y | Y | Y | 88.9 | |  |
| Malik and Razig, [20] | Y | Y | Y | | U | U | U | U | U | N | 33.3 | |  |
| Hajat and Shather, [7] | Y | N | Y | | Y | Y | Y | Y | Y | U | 77.8 | |  |
| Alkaabi et al. [24] | Y | Y | U | | Y | Y | Y | Y | Y | U | 77.8 | |  |
| Al Dhaheri, et al. [25] | Y | Y | Y | | Y | Y | Y | Y | Y | Y | 100 | |  |
| Awadallah et al., [26] | Y | U | U | | Y | Y | Y | Y | Y | U | 66.7 | |  |
| Harfil et al., [29] | Y | U | Y | | Y | Y | Y | Y | Y | U | 77.8 | |  |
| Mezhal et al., [70] | Y | Y | Y | | Y | Y | Y | Y | Y | U | 88.9 | |  |
| AlMuhairi [28] | Y | N | Y | | Y | Y | Y | Y | Y | N | 77.8 | |  |
| Mahmoud & Sulaiman, [8] | Y | Y | Y | | Y | Y | Y | Y | Y | Y | 100 | |  |
| Aziz et al., [30] | Y | Y | Y | | Y | Y | Y | Y | Y | U | 88.9 | |  |
| **Self-reported Physical activity** | | | | | | | | | | | |  | |
| Amine and Samy [31] | Y | Y | Y | | Y | N | N | N | U | U | 44.4 | |  |
| Sabri et al., [32] | Y | Y | Y | | Y | Y | Y | Y | Y | Y | 100 | |  |
| Carter et al., [33] | Y | Y | Y | | Y | Y | U | U | Y | Y | 77.8 | |  |
| Saadi et al., [34] | Y | Y | Y | | Y | Y | U | Y | Y | Y | 100 | |  |
| Muttappallymyalil et al., [35] | U | N | N | | U | Y | U | U | U | N | 11.1 | |  |
| DHHS. [1] | Y | Y | Y | | U | Y | Y | Y | U | Y | 77.8 | |  |
| Hussain and Hassan, [36] | U | N | Y | | U | U | N | N | U | N | 11.1 | |  |
| Khan et al., [37] | U | N | U | | U | Y | U | U | Y | Y | 33.3 | |  |
| Alsafar et al. [38] | Y | U | U | | Y | U | U | N | Y | U | 33.3 | |  |
| Trainer [39] | Y | U | Y | | Y | Y | Y | Y | U | U | 66.7 | |  |
| Hashim and Al-Ali [71] | Y | U | Y | | U | Y | U | U | Y | Y | 55.6 | |  |
| Katsaiti and El Anshasy, [42] | U | U | Y | | U | U | U | U | U | U | 11.1 | |  |
| Stefanopoulou et al., [41] | Y | Y | Y | | Y | Y | Y | Y | Y | U | 88.9 | |  |
| Hoffman [43] | Y | Y | Y | | Y | Y | U | Y | Y | Y | 88.9 | |  |
| Sulaiman et al., [44] | Y | Y | Y | | Y | Y | N | U | U | Y | 66.7 | |  |
| UAE NHS [3] | Y | Y | Y | | Y | Y | Y | Y | Y | Y | 100 | |  |
| Alomar et al., [46] | Y | Y | Y | | U | U | N | N | Y | N | 44.4 | |  |
| Abuelmagd et al. [45] | Y | Y | U | | Y | Y | U | Y | Y | Y | 77.8 | |  |
| Hamoudi et al., [47] | Y | U | Y | | U | Y | N | U | Y | Y | 55.6 | |  |
| Hamid et al., [6] | Y | Y | Y | | U | Y | U | U | U | Y | 55.6 | |  |
| Al Sabbah [49] | Y | Y | Y | | Y | Y | Y | Y | Y | Y | 100 | |  |
| Pramodh [48] | U | N | Y | | Y | Y | U | U | Y | U | 44.4 | |  |
| DHHS. [2] | Y | Y | Y | | Y | Y | Y | Y | Y | Y | 100 | |  |
| Hashim et al., [61] | U | Y | U | | U | U | Y | Y | U | U | 33.3 | |  |
| Bawady et al., [50] | Y | U | Y | | Y | U | U | U | U | U | 33.3 | |  |
| Sadeq et al., [51] | Y | Y | Y | | Y | Y | U | U | Y | Y | 77.8 | |  |
| Gariballa et al. [52] | Y | U | Y | | U | Y | Y | Y | Y | U | 66.7 | |  |
| AlBlooshi et al. [53] | Y | U | Y | | Y | Y | U | U | Y | U | 55.6 | |  |
| Sharma et al., [55] | Y | U | Y | | Y | Y | U | U | Y | Y | 66.7 | |  |
| Mohammad et al., [54] | Y | N | U | | Y | Y | U | U | Y | N | 44.4 | |  |
| Mezhal et al., [27] | Y | U | Y | | Y | Y | Y | Y | Y | Y | 88.9 | |  |
| Smail [56] | Y | Y | N | | Y | Y | U | N | Y | U | 55.6 | |  |
| Abd Alsamad et al., [57] | Y | Y | U | | Y | Y | U | U | Y | N | 55.6 | |  |
| Bashier et al. [58] | Y | Y | Y | | Y | Y | U | N | Y | U | 66.7 | |  |
| Almukhattin et al. [59] | Y | U | Y | | Y | Y | Y | U | Y | N | 66.7 | |  |
| **Self-reported physical activity using validated surveys** | | | | | | | | | | | |  | |
| Stubbs et al., [60] | Y | Y | U | | N | Y | Y | U | Y | Y | 66.7 | |  |
| Radwan et al., [63] | Y | N | Y | | Y | Y | Y | Y | Y | U | 77.8 | |  |
| Hashim et al., [40] | Y | U | Y | | Y | Y | Y | Y | U | U | 66.7 | |  |
| Abd-Allatif et al., [62] | Y | Y | Y | | Y | Y | Y | Y | Y | Y | 100 | |  |
| Shah et al., [64] | Y | Y | Y | | Y | Y | Y | Y | Y | Y | 100 | |  |
| Radwan et al., [67] | Y | U | Y | | Y | Y | Y | Y | Y | U | 77.8 | |  |
| Mohamedali et al. [66] | Y | U | Y | | Y | Y | Y | Y | Y | U | 77.8 | |  |
| **Physical activity using Accelerometer** | | | | | | | | | | | |  | |
| Dalibalta et al. [14] | Y | Y | Y | | Y | Y | Y | Y | Y | Y | 100 | |  |
| Qadah et al., [68] | Y | N | Y | | Y | Y | Y | Y | U | U | 66.7 | |  |
| % | 87.9 | 51.7 | 81 | | 74.1 | 84.5 | 56.9 | 56.9 | 77.6 | 38 |  | |  |

**Table S1:** N, No; U, Unclear; Y, Yes. Prespecified domains for quality assessment: a) participants (questions 1, 2, 4, and 9);

b) outcome measurement (questions 6 and 7); and c) statistics (questions 3, 5, and 8).
